# Genome-Wide Metabolic Reconstruction of the Synthesis of Polyhydroxyalkanoates from Sugars and Fatty Acids by *Burkholderia* Sensu Lato Species

**DOI:** 10.3390/microorganisms9061290

**Published:** 2021-06-12

**Authors:** Natalia Alvarez-Santullano, Pamela Villegas, Mario Sepúlveda Mardones, Roberto E. Durán, Raúl Donoso, Angela González, Claudia Sanhueza, Rodrigo Navia, Francisca Acevedo, Danilo Pérez-Pantoja, Michael Seeger

**Affiliations:** 1 Laboratorio de Microbiología Molecular y Biotecnología Ambiental, Departamento de Química & Centro de Biotecnología Daniel Alkalay Lowitt, Universidad Técnica Federico Santa María, Avenida España 1680, 2390123 Valparaíso, Chile; natalia.asg@gmail.com (N.A.-S.); sepulvedamardonesm@gmail.com (M.S.M.); ro.duran.vargas@gmail.com (R.E.D.); agonzalez.s230@gmail.com (A.G.); 2 Programa Institucional de Fomento a la Investigación, Desarrollo e Innovación (PIDi), Universidad Tecnológica Metropolitana, 8330378 Santiago, Chile; pamela.villegas@gmail.com (P.V.); radonoso@uai.cl (R.D.); danilo.perez@utem.cl (D.P.-P.); 3 Center of Applied Ecology and Sustainability (CAPES), 8330378 Santiago, Chile; 4 Scientific and Technological Bioresource Nucleus (BIOREN), Universidad de La Frontera, Casilla 54-D, 48811230 Temuco, Chile; c.sanhueza07@ufromail.cl (C.S.); rodrigo.navia@ufrontera.cl (R.N.); francisca.acevedo@ufrontera.cl (F.A.); 5 Center of Excellence in Translational Medicine (CEMT), Department of Basic Sciences, Faculty of Medicine, Universidad de La Frontera, Casilla 54-D, 48811230 Temuco, Chile; 6 Department of Chemical Engineering & Centre for Biotechnology and Bioengineering (CeBiB), Faculty of Engineering and Sciences, Universidad de La Frontera, Casilla 54-D, 48811230 Temuco, Chile

**Keywords:** polyhydroxyalkanoate, *Paraburkholderia*, *Burkholderia*, *Caballeronia*, *Trinickia*, *Micetohabitans*, *Robbsia*, PHA synthase, PHA metabolism, comparative genomics

## Abstract

*Burkholderia* sensu lato (s.l.) species have a versatile metabolism. The aims of this review are the genomic reconstruction of the metabolic pathways involved in the synthesis of polyhydroxyalkanoates (PHAs) by *Burkholderia* s.l. genera, and the characterization of the PHA synthases and the *pha* genes organization. The reports of the PHA synthesis from different substrates by *Burkholderia* s.l. strains were reviewed. Genome-guided metabolic reconstruction involving the conversion of sugars and fatty acids into PHAs by 37 *Burkholderia* s.l. species was performed. Sugars are metabolized via the Entner–Doudoroff (ED), pentose-phosphate (PP), and lower Embden–Meyerhoff–Parnas (EMP) pathways, which produce reducing power through NAD(P)H synthesis and PHA precursors. Fatty acid substrates are metabolized via *β*-oxidation and de novo synthesis of fatty acids into PHAs. The analysis of 194 *Burkholderia* s.l. genomes revealed that all strains have the *phaC*, *phaA,* and *phaB* genes for PHA synthesis, wherein the *phaC* gene is generally present in ≥2 copies. PHA synthases were classified into four phylogenetic groups belonging to class I II and III PHA synthases and one outlier group. The reconstruction of PHAs synthesis revealed a high level of gene redundancy probably reflecting complex regulatory layers that provide fine tuning according to diverse substrates and physiological conditions.

## 1. Introduction

Global plastic production reached 359 million tons in 2018, wherein 67.5% of the plastics are non-recycled, entering and polluting ecosystems [1,2]. Biodegradable bioplastics research and development have been focused towards replacing the fossil fuel-based plastics. Polyhydroxyalkanoates (PHAs) are attractive biopolymers due to their physicochemical properties, their sustainable life cycle and their wide range of applications [3,4,5,6,7]. Bacteria may accumulate PHAs as intracellular granules, especially under high carbon availability, and nitrogen, phosphorus or oxygen limitation [8,9,10]. The PHAs are classified into short chain-length PHA (PHA_scl_), with monomers with from 3 to 5 carbon chain lengths, and medium chain-length PHA (PHA_mcl_), with monomers from 6 to 14 carbons. PHA_scl_ are crystalline, stiff and brittle, whereas PHA_mcl_ are flexible and possess lower crystallinity and tensile strength [11,12,13]. Industrial production of PHAs is still limited due to the elevated price (3.5 USD/kg), which is 3-fold more expensive than conventional plastics such as polypropylene (1.2–1.3 USD/kg) [14]. This limitation has been addressed through the search of novel bacterial strains with increased PHA productivity, low-cost substrates, and bioprocess optimization [3,15].

PHA_scl_ and PHA_mcl_ polymers are synthesized from precursors that are produced from: (i) sugars through metabolic pathways such as Entner–Doudoroff (ED), pentose phosphate (PP) and Embden–Meyerhof–Parnas (EMP) pathways, and (ii) fatty acids through *β*-oxidation or de novo synthesis [16,17,18]. The PHA_scl_ polyhydroxybutyrate (PHB) is synthesized by the condensation of two molecules of acetyl-CoA by 3-ketothiolase PhaA into acetoacetyl-CoA, with a subsequent reduction by NADPH dependent acetoacetyl-CoA reductase PhaB into (*R*)-3-hydroxybutyryl-CoA (*R*-3HB-CoA). Finally, the PHA synthase PhaC polymerizes *R*-3HB-CoA into PHB [12,19]. The PHA synthases are classified in four classes. Class I, class III, and class IV PHA synthases are involved mainly in PHA_scl_ synthesis, whereas class II PHA synthases polymerize PHA_mcl_ [20]. Bacteria belonging to more than 90 genera produce PHAs [12]. Therefore, the search of bacterial metabolic networks and the genes involved in the PHA synthesis is a challenge [21,22,23].

Strains belonging to the *Burkholderia* sensu lato (s.l.) genera have been studied for their PHAs production capabilities. This group is referred as the species previously classified within the *Burkholderia* genus, and currently recognized as seven distinctive genera. The *Burkholderia* s.l. clade was divided in 2014 into the genus *Paraburkholderia*, which includes principally environmental strains, and the emended *Burkholderia* sensu stricto genus, which comprises mainly clinical and phytopathogenic strains [24]. Further phylogenetic analyses proposed a new genus, *Caballeronia*, whose species grouped as the outlined clade IIa of the *Paraburkholderia* genus [25]. Other species identified as outliers within the *Burkholderia* and *Paraburkholderia* genera have been also reclassified into *Trinickia*, *Mycetohabitans* and *Robbsia* genera [26]. Recently, another taxon has been described within *Burkholderia* s.l., the genus *Pararobbsia* [27]. Bacteria of the *Burkholderia* s.l. group possess generally large genomes (>6 Mbp) ranging from 3.7 to 11.5 Mbp and a G+C content between 58.5% to 68.5 mol%. Notably, strains belonging to these genera are adapted to a wide range of adverse environments and are metabolically versatile [28,29,30,31]. *Burkholderia* s.l. strains have been applied in bioremediation of pollutants, biocontrol of plant pathogens, plant-growth promotion, and synthesis of PHAs, enzymes, and siderophores [28,32,33,34,35,36,37,38,39,40]. The PHA synthesis by *Burkholderia* s.l. strains including *Paraburkholderia sacchari* LMG 19450^T^, *Paraburkholderia xenovorans* LB400^T^, *Burkholderia cepacia* ATCC 17759, *Burkholderia thailandensis* E264^T^, *Trinickia caryophylli* DSM 50341^T^ and *Trinickia caryophylli* AS 1.2741 has been described, suggesting an intrinsic capability within this group to produce these biodegradable polymers [5,8,39,40,41,42,43]. *Paraburkholderia*, *Burkholderia* and *Trinickia* strains use a wide range of substrates, including carbohydrates and fatty acids, to generally synthesize PHA_scl_ but also PHA_scl-mcl_ copolymers [44,45]. The aims of this review are an extensive genomic-wide reconstruction of the metabolic pathways involved in the conversion of sugars and fatty acids into the synthesis of PHAs by *Burkholderia* s.l. representative and type strains, along with a genomic-based characterization of their PHA synthases and the organization of the *pha* genes.

## 2. Synthesis of PHAs by *Burkholderia* Sensu Lato Strains

A literature search was carried out to identify *Paraburkholderia*, *Burkholderia*, *Caballeronia*, *Trinickia*, *Mycetohabitans* and *Robbsia* strains applied for PHA synthesis. Data was gathered through the search in Web of Science (Clarivate Analytics, Philadelphia, PA, USA) using keywords such as “polyhydroxyalkanoate” “polyhydroxybutyrate”, “poly(3-hydroxybutyrate)” along with “*Burkholderia*”, “*Paraburkholderia*”, “*Caballeronia*”, “*Trinickia*”, “*Mycetohabitans*”, “*Robbsia*” with the addition of other synonyms from the period 1978–2019 (data retrieved on 9 December 2019).

The most reported PHA synthesized by *Paraburkholderia*, *Burkholderia* and *Trinickia* strains is PHB. The production of PHAs by *Caballeronia*, *Mycetohabitans* and *Robbsia* strains has not been reported. *P. sacchari* LMG 19450^T^, *B. cepacia* ATCC 17759 and *B. thailandensis* E264^T^ showed to be relevant strains in PHA production (Table 1). However, PHA production has been also characterized in *P. xenovorans* LB400^T^, *B. cepacia* IPT 048, *Burkholderia* sp. F24, *Burkholderia* sp. AIU M5M02, *B. contaminans* IPT 553 and *T. caryophylli* strains DSM 50341^T^ and AS 1.2741.

### 2.1. PHB Homopolymer Synthesis by Burkholderia Sensu Lato

PHB accumulation is promoted under nutrient limitation and high levels of carbon sources [15]. PHB production in *Paraburkholderia*, *Burkholderia* and *Trinickia* has been studied mainly under nitrogen limitation (Table 1). Interestingly, under phosphorus limitation higher PHB synthesis by *P. sacchari* LMG 19450^T^ was observed than under nitrogen limitation [8]. *B. thailandensis* E264^T^, which was isolated from a rice soil in Central Thailand, is capable to synthesize PHA under nutrient balanced conditions [43].

Sugars are the most used carbon sources for the synthesis of PHB, although fatty acids have also been reported (Table 1). *B. thailandensis* E264^T^ synthesizes PHB from used cooking oil (UCO), while strain LMG 19450^T^ also produces PHB in presence of unsaturated fatty acid as co-substrates [43,44]. Interestingly, *T. caryophylli* DSM 50341^T^ produces PHB using gluconate or octanoate as the sole carbon source [46]. Glucose, gluconate, xylose, arabinose, mannitol, sucrose and fructose have been used for PHA production (Table 1). *P. sacchari* LMG 19450^T^ grown in glucose, sucrose and arabinose exhibited similar PHA production (4.0–4.2 g/L), while xylose reached a lower value (2.8 g/L). Conversely, *B. cepacia* ATCC 17759 displayed similar PHA production using glucose, fructose, sucrose (1.5–2.1 g/L) and xylose (1.5 g/L). The highest PHA yields were observed by *B. thailandensis* E264^T^ from fatty acids of used cooking oil (0.35) and by *P. sacchari* LMG 19450^T^ from sucrose (0.29), glucose (0.25–0.29) and arabinose (0.24). The differences in PHA production and PHA yield could be partly attributed to genetic determinants that are analyzed in this review.

**Table 1:** PHA homopolymers and copolymers synthesized by *Paraburkholderia* and *Burkholderia* strains.

Strain	Substrate	CDW (g/L)	PHA Type	PHA Concentration (g/L)	Limitation	Y_PHA/S_ (g/g)	Reference
*Paraburkholderia sacchari* LMG 19450^T^ (IPT 101, DSM 17165, LFM 101, CCT 6971)	Glu	5.0–6.4	PHB	0.35–4.0	Nitrogen	0.25–0.29	[14,41,47]
	Xyl	2.9–6.3	PHB	0.49–2.8	Nitrogen	0.05–0.26	[8,14,41,47,48]
	Ara	7.4	PHB	0.5–4.7	Nitrogen	0.24	[14,47]
	Man	6.9	PHB	4.2	Nitrogen	0.21	[49]
	Gal	4.9	PHB	2.2	Nitrogen	0.11	[49]
	Scr	6.14	PHB	4.2	Nitrogen	0.29	[50]
	Glu/Fatty acids	1.25–2.4	P(3HB-*co*-3HV)	0.4–0.9	-	-	[44]
	Glu/GBL, 4HBA	1.8–6.6	P(3HB-*co*-4HB)	0.4–3.1	Nitrogen	0.01–0.1 **	[44,51,52]
	Glu/HxA	2.1	P(3HB-*co*-3HHx)	1.1	Nitrogen	-	[44]
*Paraburkholderia xenovoran*s LB400^T^	Glu	-	PHB	(40% *w*/*w*)	Nitrogen	-	[40,53]
	Xyl	-	PHB	NR	Nitrogen	-	[54]
	Man	-	PHB	NR	Nitrogen	-	
*Burkholderia cepacia* ATCC 17759 (DSM 50181)	Glu	2.6	PHB	1.5	Nitrogen	-	[55]
	Fru	5	PHB	2	Nitrogen	0.07–0.174	[55,56]
	Xyl	2.6	PHB	1.5	Nitrogen	0.11	[55]
	Scr	4.2	PHB	2.1	Nitrogen	0.18	[50]
	Xyl/LaA	5.3	P(3HB-*co*-3HV)	2.4	-	-	[57]
	Glu/PA	1.6–1.8	P(3HB-*co*-3HV)	0.2–1.0	-	-	[56]
*Burkholderia thailandensis* E264^T^	UCO (fatty acids)	12.6	PHB	7.5	NL	0.35	[43]
*Burkholderia contaminans* Kad1	Waste glycerol/VA	5.6	P(3HB-*co*-3HV)	1.96	-	-	[58]
*Burkholderia contaminans* IPT 553	Glu/Scr	2.3–4.9	P(3HB-*co*-3HDd)	0.85–1.176	-	-	[45]
*Trinickia caryophylli* DSM 50341^T^	Gnt	-	PHB	(34.2% *w*/*w*)	-	-	[46]
	OA	- *	PHB	1.2	-	-	
*Trinickia caryophylli* AS 1.2741	Glu	0.981	P(3HHx-*co*-3HO-*co*-3HD)	0.013	-	-	[59]
	OA	1.084	P(3HHx-*co*-3HO-*co*-3HD)	0.26	-	-	
	Glu/OA	1.159	P(3HHx-*co*-3HO-*co*-3HD)	0.23	-	-	

Glu, glucose; Xyl, xylose; Ara, arabinose; Man, mannitol; Gal, galactose; Scr, sucrose; Gnt: gluconate; Fatty acids: propionic; valeric (VA), heptanoic, nonanoic, undecanoic acid; 3HB: 3-hydroxybutyryl; 3HV: 3-hydroxyvaleryl; 3HDd: 3-hydroxydodecanoyl; GBL: gamma-butyrolactone; 4HBA: 4-hydroxybutyic acid; HxA: hexanoic acid; Fru, fructose; LaA: Lauric acid; PA, propionic acid; OA, octanoic acid; P(3HB-*co*-3HV), poly(3-hydroxybutyrate-*co*-3-hydroxyvalerate); P(3HB-*co*-4HB), poly(3-hydroxybutyrate-*co*-4-hydroxybutyrate); P(3HB-*co*-3HHx), poly(3-hydroxybutyrate-*co*-3-hydroxyhexanoate); P(3HB-*co*-3HDd), poly(3-hydroxybutyrate-*co*-3-hydroxydodecanoate); P(3HHx*-co-*3HO-*co*-3HD), poly(3-hydroxyhexanoate-*co*-3-hydroxyoctanoate-*co*-3-hydroxydecanoate); UCO: used cooking oil. CDW: cell dry weight; NR: not reported; NL: not limited; * no growth reported, ** g P4HB/g GBL.

### 2.2. PHA Copolymer Synthesis by Burkholderia Sensu Lato

The PHA copolymers synthesized by *Paraburkholderia*, *Burkholderia* and *Trinickia* strains are composed of PHA_scl_ and PHA_mcl_ monomers. Different metabolic pathways can supply the intermediates for PHA copolymers. *P. sacchari* LMG 19450^T^ produces the copolymer poly(3-hydroxybutyrate-*co*-3-hydroxyvalerate) (P(3HB-*co*-3HV)) from glucose and odd-chain fatty acids as co-substrates, increasing 3-hydroxyvaleryl (3HV) content when valeric acid is supplied as co-substrate [44]. The 3HV content decreases with longer odd-chain fatty acids supplied, due to the higher level of acetyl-CoA generated through *β*-oxidation, which increased 3HB monomer synthesis. However, when even-numbered saturated or unsaturated fatty acids are supplied as co-substrates, strain LMG 19450^T^ produces only PHB [44]. The synthesis of P(3HB-*co*-3HV) by *Paraburkholderia* and *Burkholderia* strains fed with glucose, succinate, xylose and glycerol in presence of a co-substrate such as levulinic acid (LA), valeric acid and propionic acid has been reported (Table 1). LA may be obtained by acid catalysis of low-cost renewable sources, including cellulose-forest and agricultural residues, therefore, is an interesting co-substrate for PHA production in bacteria resistant to the LA‒related toxicity [60,61]. Strains *P. sacchari* LMG 19450^T^ and *B. contaminans* IPT 553 produce the PHA_scl-mcl_ co-polymer poly(3-hydroxybutyrate-*co*-hydroxyhexanoate) (P(3HB-*co*-3HHx)) from related and unrelated substrates, respectively (Table 1). *T. caryophylli* AS 1.2741 strain produces the PHA_mcl_ copolymers poly(3-hydroxyhexanoate-*co*-3-hydroxyoctanoate-*co*-3-hydroxydecanoate) (P(3HHx-*co*-3HO-*co*-3HD)) from different substrates [59].

The copolymers composed of 3HB and 4-hydroxybutyrate P(3HB-*co*-4HB) possess improved physical, thermal and biodegradability properties compared to poly-3-hydroxybutyrate (P(3HB)) [62]. The 4HB precursor is generated through the oxidation of 1,4-butanediol by an alcohol dehydrogenase and an acetaldehyde dehydrogenase into 4-hydroxybutanoate, which is transformed by a hydroxyacyl-CoA synthase into 4-hydroxybutyryl-CoA [23]. *P. sacchari* LMG 19450^T^ produces P(3HB-*co*-4HB) from γ-butyrolactone (GBL) that is converted into 4-hydroxybutanoate, reaching concentrations of the copolymer of 37 g/L with 5% 4HB monomer [51]. 4-Hydroxybutanoate is partially converted into 4-hydroxybutyryl-CoA but is mainly oxidized into succinic acid semialdehyde and succinic acid, which is further converted to acetyl-CoA and then into 3-hydroxybutyryl-CoA [51].

The PHA precursors provided by diverse catabolic pathways of different substrates have been related to PHA productivity and monomeric composition [21,63,64,65]. However, the diversity of metabolic pathways and genetic determinants related to PHA synthesis in *Burkholderia* s.l. bacteria have only partly been reported [66].

## 3. Metabolism of Sugars and Fatty Acids in *Burkholderia* Sensu Lato

At March 2021, more than 150 validly published species represent the *Burkholderia* s.l. group, including: 78 *Parabukholderia* spp., 34 *Burkholderia* spp., 27 *Caballeronia* spp., 7 *Trinickia* spp., 2 *Mycetohabitans* spp., 1 *Robbsia* spp., and 2 *Pararobbsia* spp. [67]. Based on the proportion of species described in *Burkholderia* s.l., and identifying representative clades within each genus, a genome selection was carried out for further analysis. Selection was performed using AnnoTree [68], the web browser based on taxonomy information derived from the Genome Taxonomy Database phylogeny [69]. *Burkholderia* s.l. species were further selected based on their phylogenomic placement at the infrageneric level, the availability of a metabolic network in curated databases [70] or a genome sequence in public databases. The metabolism of 37 selected strains of *Burkholderia* s.l., each belonging to a different species, was analyzed to identify genetic determinants and pathways involved in the conversion of sugars, fatty acids and related compounds for the production of PHAs. The selection consisted of 13 *Burkholderia*, 14 *Paraburkholderia*, 6 *Caballeronia*, 2 *Trinickia*, 1 *Mycetohabitans* and 1 *Robbsia* genomes (Table 2). *Pararobbsia* strains were not analyzed in this review as both species comprised in the genus, *Pararobbsia alpina* and *Pararobbsia silviterrae*, are newly proposed species with no relevant information besides their species description.

**Figure 1:**
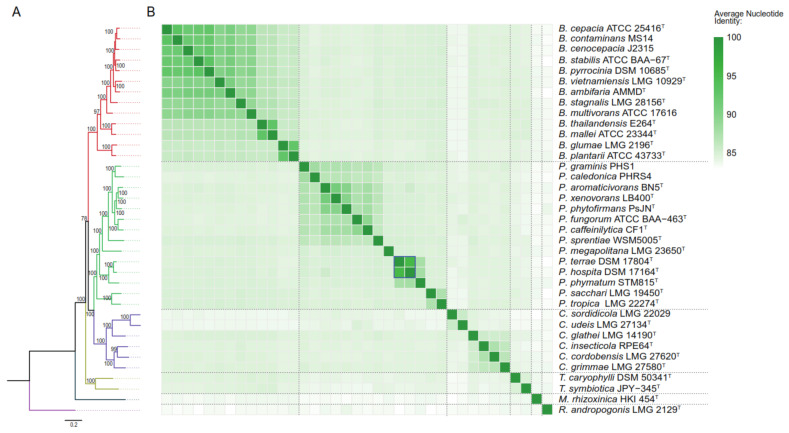
Phylogenomic placement of the 37 *Burkholderia* sensu lato genomes used in this review. (**A**), phylogenomic tree of *Burkholderia* sensu lato strains based on 38 core genes (Phylophlan software); (**B**), Average Nucleotide Identity based on Mummer (ANIm) analysis of *Burkholderia* sensu lato strains.

The evolutionary relatedness between each taxa against the genetic and metabolic traits involved in carbohydrates and fatty acids assimilation was assessed. For this purpose, a phylogenomic analysis of the 37 selected strains was conducted. A phylogeny of 38 concatenated core genes was constructed using the Phylophlan software [71], followed by a maximum-likelihood analysis. The bootstrap confidence values were calculated with 1000 replicates, while values below 50% were not shown (Figure 1A). As expected, six clades were clearly distinguished, each of them representing one genus belonging to *Burkholderia* s.l. (*Burkholderia* sensu stricto, red branch; *Paraburkholderia* green branch; *Caballeronia*, purple branch; *Trinickia*, yellow branch; *Mycetohabitans*, dark blue branch; and *Robbsia*, pink branch; Figure 1A). To further corroborate each species placement, an average nucleotide identity based on Mummer (ANIm) analysis was conducted. Genomic index values supported the clades identified by the Phylophlan software (Figure 1B). Among the 37 *Burkholderia* s.l. genomes analyzed, ANIm values were below the current cut-off for species delineation (>95–96%, Richter and Roselló-Mora, 2009), excluding *Paraburkholderia hospita* DSM 17164^T^ and *Paraburkholderia terrae* DSM 17804^T.^ (ANIm 95.4%, blue square, Figure 1B). The taxonomic classification of the *Paraburkholderia* subgroup was assessed by Pratama et al., obtaining the same ANIm value (95.42%) when comparing *P. terrae* and *P. hospita* type strains. The authors proposed a larger species “cluster”, represented by *P. hospita* and supported by phylogeny based on 16S rRNA gene sequence, multilocus sequence analysis using 7 concatenated genes, ANIm, tetranucleotide frequencies (TETRA), and comparative genomics [72].

**Figure 2:**
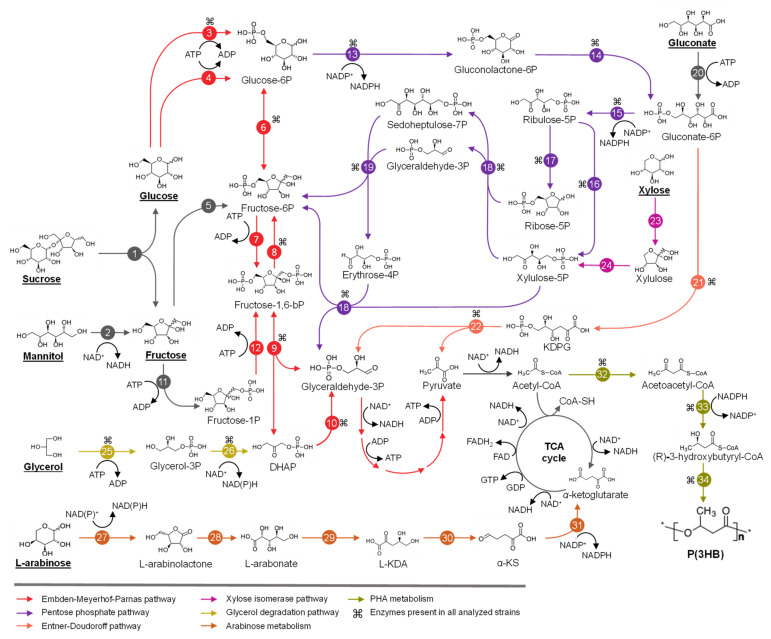
Proposed metabolic pathways from sugars and glycerol into the synthesis of polyhydroxybutyrate of *Burkholderia* sensu lato genera. Each reaction is represented with an arrow with the respective enzyme depicted with a number. Compounds with their name underlined are carbon and energy sources for these bacteria. Enzymes present in all analyzed genomes are marked with “⌘”.1, β-fructofuranidase (FFase), α-glucosidase (MalL); 2, mannitol dehydrogenase (MDH); 3, glucokinase (GLK); 4, glucose phosphoenolpyruvate transferase system (PTS) family transporter (glu-EII); 5, fructokinase (FK); 6, glucose-6-phosphate (G6P) isomerase (G6PI); 7, 6-phosphofructokinase (6PFK); 8, fructose-1,6-phosphatase (FBPase); 9, fructose-1,6-biphosphate aldolase (FBA); 10 triosephosphate isomerase (TIM); 11, Fructose PTS family transporter (fru-EII); 12, 1-phosphofructokinase (1PFK); 13, G6P dehydrogenase (G6PDH); 14, 6-phosphogluconolactonase (6PGnL); 15, phosphogluconate dehydrogenase (6PGntD); 16, ribulose-5-phosphate epimerase (R5PE); 17, ribulose-5-phosphate isomerase (R5PI); 18, transketolase (TKT); 19, transaldolase (TAL); 20, gluconate kinase (GntK); 21, phosphogluconate dehydratase (PGntDT); 22, 2-keto-3-deoxyphosphogluconate (KDPGnt) aldolase (KDPGA); 23, xylose isomerase (XI); 24, xylulokinase (XK); 25, glycerol kinase (GK); 26, glycerol-3-phosphate dehydrogenase (G3PDH); 27, L-arabinose1-dehydrogenase (LADH); 28, arabinolactonase (AL); 29, arabonate dehydratase (AD); 30, L-KDA dehydratase (LKDADT); 31, α-ketoglutarate semialdehyde dehydrogenase (KGSADH); 32, ketothiolase (PhaA); 33, acetoacetyl-CoA reductase (PhaB); 34, PHA synthase (PhaC). DHAP: dihydroxyacetone phosphate; α-KS: α-ketoglutarate semialdehyde; TCA; tricarboxylic acid cycle. Homology prediction of 27 strains was performed using the curated metabolic networks of the Kyoto Enzyme and Genomes Database (KEGG) and manual Blast search through Bidirectional Best Hit approach (BBH). For the 10 strains belonging to novel genera (e.g., *Caballeronia*, *Trinickia*, *Mycetohabitans*, *Robbsia*) a manual reconstruction through BBH was performed. An amino acid sequence identity of >30% and ≥70% coverage was used as threshold in function of the gene context for homology prediction. Genomes were retrieved from Refseq database.

**Figure 3:**
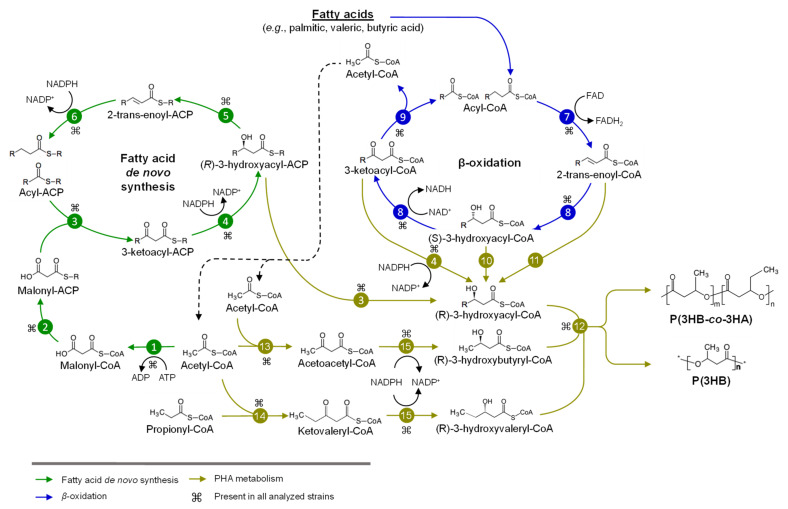
Proposed metabolism of fatty acids associated to the synthesis of PHAs in *Burkholderia* sensu lato genera. Each reaction is represented with an arrow, with the respective enzyme or enzymes that catalyze the reaction depicted with a number. Enzymes present in all genomes are marked with “⌘”. 1, acetyl-CoA carboxylase (ACC); 2, malonyl-CoA-ACP transacylase (MAT); 3, class I-III β-ketoacyl-ACP synthases (KAS); 4, 3-ketoacyl-ACP reductase (KR); 5, β-hydroxyacyl-ACP-dehydratase (HAD); 6, enoyl-ACP reductase (ENR); 7, acyl-CoA dehydrogenase (ACAD); 8, multifunctional S-specific enoyl-CoA hydratase-hydroxyacyl-CoA dehydrogenase (HCDH/ECH); 9, β-ketoacyl-CoA thiolase (KAT); 10, 3-hydroxybutyryl-CoA epimerase (HB3E); 11, R-specific enoyl-CoA hydratase (R-ECH); 12, PHA synthase (PhaC); 15, β-ketothiolase (PhaA); 16, β-ketothiolase (BktB); 17, acetoacetyl-CoA reductase (PhaB). P(3HB-*co*-3HA), poly(3-hydroxybutryrate-*co*-3-hydroxyacyl) represents any copolymer containing PHA_scl_ monomers (e.g., 3-hydroxybutyrate) and PHA_mcl_ monomers (e.g., 3-hydroxyacyl). Homology prediction of 27 strains was performed using the curated metabolic networks of the Kyoto Enzyme and Genomes Database (KEGG) and manual Blast search through Bidirectional Best Hit approach (BBH). For the 10 strains belonging to novel genera (e.g., *Caballeronia*, *Trinickia*, *Mycetohabitans*, *Robbsia*) a manual reconstruction through BBH was performed. An amino acid sequence identity of >30% and ≥70% coverage was used as threshold in function of the gene context for homology prediction. Genomes were retrieved from Refseq database.

For the identification of genetic determinants involved in the metabolism of sugars and fatty acids, genome-based metabolic networks were retrieved from the Kyoto Encyclopedia of Genes and Genomes (KEGG) database. For the strains without a prior network (e.g., *Caballeronia* spp., *T. caryophylli* DSM 50341^T^, *Trinickia symbiotica* JPI-345^T^, *Robbsia andropogonis* LMG 2129^T^) a genome-based reconstruction was performed manually by a Bidirectional Best Hit (BBH) approach. Absent enzymes from the metabolic networks (e.g., PhaG, PhaJ, GlK, GlpD, BktB) were manually searched through a BBH approach, against amino acid sequences with experimental evidence obtained from the Swiss-Prot database, using a threshold of ≥30% identity and ≥70% coverage. For the identification of each genetic determinant, the genomic context was analyzed, and all reactions reported in the present study were manually curated and depicted in Figure 2 and Figure 3.

**Figure 4:**
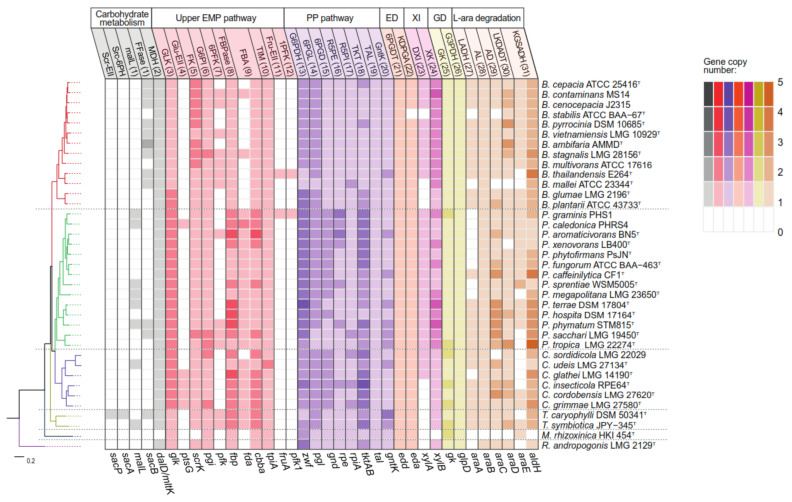
Genomic comparison of the metabolism for the conversion of sugars and glycerol into PHA by *Burkholderia* sensu lato (s.l.) strains. Enzymes functions and their respective coding genes are listed above and below the graph, respectively. Numbers shown in parenthesis following the enzyme activity correspond to reactions represented in Figure 2. Color intensity depicts the gene copy number as showed in legend. Homology prediction of 27 strains was performed using the curated metabolic networks of the Kyoto Enzyme and Genomes Database (KEGG) and manual Blast search through Bidirectional Best Hit approach (BBH). For the 10 strains belonging to novel genera (e.g., *Caballeronia*, *Trinickia*, *Mycetohabitans*, *Robbsia*) a manual reconstruction through BBH was performed. An amino acid sequence identity of >30% and ≥70% coverage was used as threshold in function of the gene context for homology prediction. Genomes were retrieved from Refseq database. Strains are arranged according to a phylogenomic tree constructed from a concatenate of 38 core genes present in the 37 genomes using Phylophlan and MAD root softwares. Scr-EII, sucrose transporter of the phosphoenolpyruvate system (PTS) family; Scr-6PH, sucrose-6-phosphate hydrolase; MalL, α-glucosidase; FFase, β-fructofuranidase, MDH, mannitol dehydrogenase; GLK, glucokinase; glu-EII, glucose PTS family transporter; FK, fructokinase; G6PI, glucose-6-phosphate (G6P) isomerase; 6PFK, 6-phosphofructokinase; FBPase, fructose-1,6-phosphatase; FBA fructose-1,6-biphosphate aldolase; TIM, triosephosphate isomerase; fru-EII, Fructose PTS family transporter; 1PFK, 1-phosphofructokinase; G6PDH, G6P dehydrogenase; 6PGL, 6-phosphogluconolactonase; 6PGntD, phosphogluconate dehydrogenase; R5PE, ribulose-5-phosphate epimerase; R5PI, ribulose-5-phosphate isomerase; TKT, transketolase; TAL, transaldolase; GntK, gluconate kinase; PGDT, phosphogluconate dehydratase; KDPGntA, 2-keto-3-deoxyphosphogluconate aldolase; XI, xylose isomerase; XK, xylulokinase; GK, glycerol kinase; G3PDH, glycerol-3-phosphate dehydrogenase; LADH, L-arabinose-1-dehydrogenase; AL, arabinolactonase; AD, arabonate dehydratase; LKDADT, L-KDA dehydratase; KGSADH, α-ketoglutarate semialdehyde dehydrogenase.

**Figure 5:**
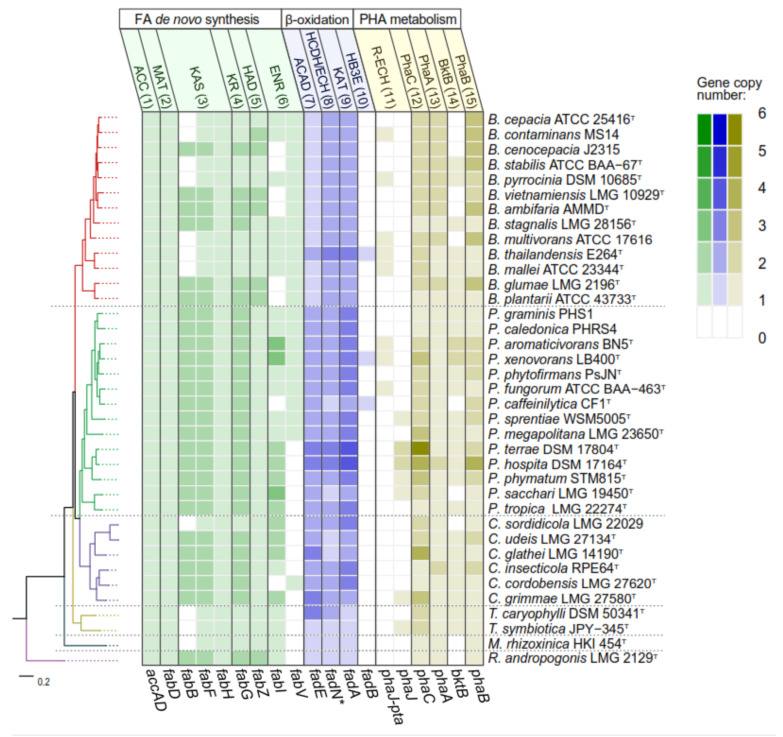
Genomic comparison of the metabolism for the conversion of fatty acids into PHA by *Burkholderia* sensu lato strains. Enzymes functions and their respective coding genes are listed above and below the graph, respectively. Number shown in parenthesis following the enzyme activity correspond to reactions shown in Figure 2 and Figure 3. Color intensity depicts the gene copy number as showed in legend. Homology prediction of 27 strains was performed using the curated metabolic networks of the Kyoto Enzyme and Genomes Database (KEGG) and manual Blast search through Bidirectional Best Hit approach (BBH). For the 10 strains belonging to novel genera (e.g., *Caballeronia*, *Trinickia*, *Mycetohabitans*, *Robbsia*) a manual reconstruction through BBH was performed. An amino acid sequence identity of >30% and ≥70% coverage was used as threshold in function of the gene context for homology prediction. Genomes were retrieved from Refseq database. Strains are arranged according to a phylogenomic tree constructed from a concatenate of 38 core genes present in the 37 genomes using Phylophlan and MAD root software. ACC, acetyl-CoA carboxylase; MAT, malonyl-CoA-ACP transacylase; KAS, class I-III β-ketoacyl-ACP synthases; KR, 3-ketoacyl-ACP reductase; HAD, β-hydroxyacyl-ACP-dehydratase; ENR, enoyl-ACP reductase; ACAD, acyl-CoA dehydrogenase; HCDH/ECH multifunctional S-specific enoyl-CoA hydratase-hydroxyacyl-CoA dehydrogenase; KAT, β-ketoacyl-CoA thiolase; HB3E, 3-hydroxybutyryl-CoA epimerase; R-ECH, R-specific enoyl-CoA hydratase; PhaC, PHA synthase; PhaA, β-ketothiolase; BktB, β-ketothiolase; PhaB, acetoacetyl-CoA reductase.

In general, *Paraburkholderia* and *Caballeronia* strains show higher gene redundancy than the rest of the *Burkholderia* s.l. genera in the EMP, PP of carbohydrates metabolism (Figure 4), and *β*-oxidation and fatty acid de novo synthesis pathways of fatty acids metabolism (Figure 5). For example, in carbohydrate metabolism (Figure 4) *Paraburkholderia* and *Caballeronia* strains have 1 to 3 genes coding for glucokinase (*glk*), glucose-6-phosphate dehydrogenase (*zwf*), and transketolase (*tkt*). On the other hand, specific clades of the *Burkholderia* genus (*Burkholderia cepacia* complex) presented two copies of fructokinase *scrK* gene. Similarly, in *Paraburkholderia* and *Caballeronia* genera, more copies of the *β*-oxidation *fadE* and *fadA* genes along with fatty acid de novo synthesis *fabG* and *fabI* genes are present (Figure 5). Interestingly, the essential enzyme for unsaturated fatty acid synthesis in *E. coli*, class I ketoacyl-ACP synthase (KAS) that is encoded by the *fabB* gene, showed higher presence in *Paraburkholderia* and *Caballeronia* strains than the rest of *Burkholderia* s.l. genera [73]. On the other hand, genes related to PHA metabolism are highly variable among species, especially in the *Paraburkholderia* and *Caballeronia* genera, probably due to horizontal gene transfer events described previously [74]. In addition, five *Paraburkholderia,* two *Caballeronia* and the *Burkholderia* cepacia complex strains possess additional copies of the *phaA* and *phaB* genes encoded in two gene clusters harboring a phasin gene, the *phaPBA* cluster and *phaPB* gene cluster. Finally, *Mycetohabitans rhixorzinica* HKI 454^T^ possess different genetic profile due to the absence of fructose, xylose, sucrose, mannitol and arabinose assimilation pathways along with a low copy number in EMP, PP, *β*-oxidation and fatty acid de novo synthesis pathways. The substrate assimilation by *Burkholderia* s.l. strains was reviewed in Table S1 [75,76,77,78,79,80,81,82,83,84,85,86,87,88,89,90,91,92,93,94,95]. Remarkably *Mycetohabitans rhixorzinica* HKI 454^T^ is the only strain unable to grow on glucose as the sole carbon source.

### 3.1. Metabolism of Sugars for PHA Production in Burkholderia Sensu Lato

Sucrose, glucose, fructose, xylose, mannitol, gluconate and glycerol were the analyzed substrates for the genome-based reconstruction of metabolic pathways in *Burkholderia* s.l. strains for their conversion into PHA, as they are the main compounds reported for PHA production by these bacteria (Table 1).

In *Burkholderia* sensu stricto strains, sucrose is hydrolyzed by sucrose hydrolase β-fructofuranosidase (SacB) into glucose and fructose (Figure 2). In contrast, most of *Paraburkholderia* and *Caballeronia* strains are unable to metabolize sucrose, except for *Paraburkholderia graminis* PHS1 and *Paraburkholderia megapolitana* LMG 23650^T^ (Table S1), which probably break down sucrose through α-glucosidase (MalL). *T*. *caryophylli* DSM 50341^T^ is able to import sucrose through a phosphoenolpyruvate transferase system (PTS) family transporter, yielding sucrose-6-phosphate that is subsequently transformed to glucose-6-phosphate (G6P) and D-fructose by a sucrose-6-phosphate hydrolase. Except for *Mycetohabitans rhizoxinica* HKI 454^T^, mannitol is oxidized by mannitol dehydrogenase (MltK) or arabinitol 4-dehydrogenase (DalD) into fructose, yielding NADH equivalents. All the strains have ABC transporter genes for glucose import. Glucose is subsequently phosphorylated into G6P by glucokinase enzyme, encoded by the *glk* gene that is present in all analyzed strains. Particularly, *Paraburkholderia* and *Caballeronia* genomes possess an additional polyphosphate-dependent glucokinase gene (polyP-Glk). The closest relative of these enzymes is the polyP-Glk of *Anabaena* sp. PCC 7120, described to participate in nitrogen deprivation stress resistance in this cyanobacterium [96]. PolyP-Glk is also present in the phytopathogens *Burkholderia plantarii* ATCC 43733^T^ and *Burkholderia glumae* LMG 2196^T^ (Figure 4). Additionally, in *Caballeronia* strains, excluding *Caballeronia sordidicola* LMG 22029 and *Caballeronia udeis* LMG 27134^T^ glucose may enter through a phosphotransferase system (PTS) family transporter, producing G6P (Figure 2 and Figure 4) [97,98]. Most of the *Burkholderia* s.l. strains probably incorporate fructose by an ABC family transporter, and then fructose is phosphorylated by fructokinase into fructose-6-phosphate (F6P) (Figure 2 and Figure 4). In contrast, in *P. graminis* PHS1 and *B. thailandensis* E264^T^, fructose may be transported into the cell through a PTS system that phosphorylates fructose into fructose-1-phosphate (F1P) [99]. F6P and F1P can be phosphorylated by a 6-phosphofructokinase (Pfk, *pfk* gene) into fructose-1,6-bisphosphate (F1,6bP) and then metabolized through the glycolytic EMP pathway (Figure 2; [99])Alternatively, F1,6bP may enter the gluconeogenic EMP pathway to generate F6P that is converted by a G6P isomerase into G6P [99]. Interestingly, from all the analyzed strains, only 53% *Burkholderia*, 28% *Paraburkholderia* and both *Trinickia* genomes possessed a complete EMP pathway (Figure 2 and Figure 4). In contrast, the EMP pathway is incomplete in the rest of the analyzed strains, due to the absence of the *pfk* gene. Notably, in all the strains with complete EMP pathway, the *pfk* gene is located from 0 to12 ORFs upstream of a *phaC* gene organization. The interrupted EMP pathway due to *pfk* absence has also been reported in the *Ralstonia* genus, marine bacteria of Alphaproteobacteria, Gammaproteobacteria and Flavobacteria classes, and *Pseudomonas* strains [99,100,101,102,103,104].

In *Burkholderia* s.l. strains, G6P, obtained from sugar metabolism and gluconeogenic EMP, is oxidized in the pentose phosphate (PP) pathway by a G6P dehydrogenase (G6PDH), enzyme encoded by the *zwf* gene, yielding gluconolactone-6-phosphate (GL-6P) and NADPH. 6-Phosphogluconolactonase (6PGL) hydrolyzes GL-6P an into gluconate-6-phosphate (gluconate-6P), which is channeled through the ED or PP pathways (Figure 2). Gluconate-6P can also be obtained from gluconate by gluconokinase in *Burkholderia* s.l. strains. ED and PP pathways were complete and conserved in the *Burkholderia* s.l. strains (Figure 4). G6PDH is a key enzyme in the oxidative branch of PP pathway during glucose oxidation [105], and in *Burkholderia* s.l. strains the *zwf* gene coding for this enzyme is located in the *zwf-pgl-glk* organization. *Burkholderia*, *Caballeronia*, *Mycetohabitans* and *Trinickia* strains have up to 2 *zwf* gene copies (Figure 4), while *Paraburkholderia* and *Robbsia* strains possess a third *zwf* gene copy located next to *polyP-glk* gene and a glycogen debranching gene. The *Burkholderia* phytopathogen subgroup also possesses the *polyP-glk-zwf* arrangement. This suggests that the *zwf* gene redundancy in these bacteria may play a role during the adaptation of these organisms in environments of variable nutrient availability, as mentioned before for *polyP-glk* gene and the NADPH synthesis via the oxidative branch of PP pathway [96,105]. During the conversion of G6P into pyruvate via the ED pathway 1 molecule of NADPH, 1 molecule of NADH and 2 ATP are produced, whereas only 1 molecule of NADH but 3 ATP are synthesized through the EMP pathway. In comparison, the degradation of G6P via the PP pathway produces up to 6 molecules of NADPH, 1 molecule of NADH and 2 ATP. In *Pseudomonas*, the carbon derived from gluconate-6P is funneled through ED pathway and then through the gluconeogenic EMP and oxidative PP pathways to yield NADPH [99,100]. Furthermore, metabolic flux analysis in *Pseudomonas putida* KT2440 and *Pseudomonas protegens* Pf-5 (both strains lack the *pfk* gene) demonstrated that ~90% of glucose enters the ED pathway through gluconate-6P, in part attributed to the lower protein expenses leading to NADPH accumulation [99,101,106]. It has also been observed that the deficiency in the EMP pathway in diverse bacteria of Alphaproteobacteria, Gammaproteobacteria and Flavobacteria classes leads to an increase of NADPH supplied by the ED pathway [102,103]. In *Pseudomonas* and *Chromohalobacter* genera, the oxidative phase of PP, ED and the gluconeogenic EMP pathway generate a cycle that promotes biosynthetic precursors and NADPH equivalents [99,101,106]. When the EMP pathway is completed by the insertion of the *pfk* gene in *P. putida* KT2440 strain, this cycle may be bypassed, decreasing ATP content (~50%) and reducing NADPH/NADP+ ratio from 1.4 to ~0.6 [107]. In addition, *P. putida* KT2440 segregates the carbon provided from glucose and benzoate in the upper EMP-ED-PP cycle and tricarboxylic acid cycle (TCA cycle), respectively, in order to supply biosynthetic compounds flux and NADPH demands [100]. Similarly, *Ralstonia* species lack *pfk* and gluconate-6-phosphate dehydrogenase (*gnd*) genes, resulting in interrupted EMP and oxidative PP pathways, placing ED pathway as the main route for glucose oxidation and NADPH production, according to carbon isotope labeling studies [104]. NADPH provides reducing power to endure oxidative stress by reducing antioxidative molecules (e.g., glutathione, thioredoxin and alkyl hyperoxide reductase) and the synthesis of compounds related to stress resistance (e.g., ectoines, PHAs) [101,108,109]. Model bacteria for the degradation of aromatic compounds such as *P. xenovorans* LB400^T^ and *P. putida* KT440 possess strong antioxidative systems that avoid the accumulation of reactive oxygen species (ROS) during degradation of aromatic compounds [110,111,112]. Therefore, maintaining a high NAD(P)H/NAD(P)+ ratio could contribute to the detoxification of ROS during aromatic degradation [111]. Sacomboio et al. [113] reached a 2-fold increase in PHA production related to a 2-fold increment in the NADPH/NADP+ ratio due the increased expression of G6PDH by mutating the *ntrC* regulator in the *Burkholderiales* bacterium, *Herbaspirillum seropedicae*. The metabolic traits described in *Pseudomonas* are probably also involved in the PHA metabolism in *Burkholderia* s.l. genera. These metabolic networks may allow the adaptation under the fluctuating environmental conditions where species of *Pseudomonas* and *Burkholderia* s.l. strains inhabit [107,111,114]. However, further insights are needed to confirm a cyclic metabolic flux in EMP, ED and PP pathways that promotes PHA synthesis in *Burkholderia* s.l. bacteria.

Xylose (“wood sugar”) and arabinose are present in agricultural by-products used for PHA production [8]. In *Burkholderia* s.l. strains, xylose is isomerized to xylulose and then phosphorylated into xylulose-5-phosphate (Xyl-5P) by xylose isomerase and xylulokinase [115]. The xylose isomerase pathway is conserved in the *Paraburkholderia* and *Burkholderia* sensu stricto strains. Xyl-5P is funneled into the non-oxidative PP pathway. Xyl-5P and ribose-5-phosphate are converted by transketolase and transaldolase via sedoheptulose-7-phosphate, glyceraldehyde-3-phosphate (GlyA-3P) and erythrose-4-phosphate into F6P and GlyA-3P (Figure 2). F6P is isomerized in the gluconeogenic EMP pathway to G6P and channeled into ED pathway, yielding NADPH. PHB production using xylose as the sole carbon source have been reported in *P. sacchari* LMG 19450^T^, showing 40% lower PHB production compared to glucose [41]. This difference has been related to the higher reductive power provided by glucose compared to xylose due to the additional NADPH production by G6PDH and GL-6P dehydrogenase during glucose catabolism via oxidative PP pathway than the xylose isomerase pathway. Conversely, *B. cepacia* ATCC 17759 showed similar PHB concentration from glucose and xylose (Table 1) [55]. This may be explained by an alternative xylose degradation pathway, described previously in *Caulobacter crescentus*, which exhibit NAD(P)H generation by xylose dehydrogenase (XylA) and α-ketoglutaric semialdehyde dehydrogenase (XylC) [115]. However, this alternative catabolic pathway is incomplete since the *xylA* gene was not found in none of the analyzed strains and ATCC 17759 draft genome. Furthermore, *B. glumae* LMG 2196T*, B. plantarii* ATCC 43733T and *T. caryophylli* DSM 50341^T^ can assimilate xylose as the sole carbon source (Table S1) although both pathways are incomplete (Figure 4), suggesting the presence of an alternative xylose degradation route or that the enzymes of these pathways are distantly related. The xylose isomerase pathway is conserved in the *Paraburkholderia* genus, 9 *Burkholderia* species and 2 *Caballeronia* species (Figure 4).

PHA production from arabinose has been reported in *P. sacchari* LMG 19450^T^ [14,47] and most of the analyzed strains assimilate arabinose (Table S1). The *ara* genes of the classical arabinose catabolic pathway [116] were not found in the analyzed strains (Figure 4). This suggests a second arabinose catabolic pathway, involving non-phosphorylated metabolic intermediates [116]. L-arabinose is converted by L-arabinose 1-dehydrogenase, *L*-arabinonolactonase, and *L*-arabonate dehydratase to *L*-2-keto-3-deoxyarabonate (L-KDA) (Figure 2). L-KDA is transformed by L-KDA dehydratase into α-ketosemialdehyde (α-KS). Finally, α-KS is converted to α-ketoglutarate by the type I α-ketoglutaric semialdehyde dehydrogenase (KGSADH) enzyme encoded by the arabinose inducible *araE* gene [117]. This pathway is conserved in all *Burkholderia* sensu stricto strains except for *Burkholderia mallei* ATCC 23344^T^, *B*. *thailandensis* E264^T^ and *Burkholderia stabilis* ATCC BAA-67^T^. Conversely, 10 *Paraburkholderia*, 4 *Caballeronia*, *T*. *caryophylli* DSM 50341^T^, *M*. *rhizoxinica* HKI 454^T^ and *R. andropogonis* LMG 2129^T^ strains have an incomplete arabinose degradation pathway. One group of eight *Paraburkholderia*, *Caballeronia glathei* LMG 14190^T^ and *M. rhizorxinica* HKI 454^T^ strains lacks the *araA* gene encoding L-arabinose dehydrogenase (ADH) or other arabinose degradation genes. Interestingly all these strains assimilate this substrate, except *B. mallei* ATCC 23344^T^ (Table 1 and Table S1), suggesting an alternative arabinose assimilation pathway. On the other hand, a second group of 2 closely related *Paraburkholderia*, 4 *Caballeronia* strains and *B. thailandensis* E264^T^ lacks the *araE* gene encoding KGSADH enzyme (Figure 4) although they can assimilate *L*-arabinose (Table S1). Interestingly, all these strains possess 1−5 additional *aldH* gene copies encoding type II and III KGSADH enzymes, which are induced in *Azospirillum brasilense* by *D*-glucarate/*D*-galactarate and hydroxyproline, respectively. These KGSADH homologs encoded by the *aldH* genes probably can be induced by *L*-arabinose and complete the arabinose degradation in these strains [118].

Glycerol is assimilated by *B. cepacia* strains ATCC 17759 and IPT 438, *Burkholderia* sp. AB4 and *P. sacchari* LMG 19450^T^ for PHB synthesis [119,120]. The glycerol catabolic pathway is conserved in the *Burkholderia* s.l. strains (Figure 4). Glycerol is transported into the cell by a facilitator transporter (GlpF) and phosphorylated by glycerol kinase (GlpK) to yield glycerol-3-phosphate (G3P). G3P is transformed by glycerol-3-phosphate dehydrogenase (GlpD) to dihydroxyacetone phosphate, which is funneled into the lower EMP pathway.

### 3.2. Metabolism of Fatty Acids and PHA Synthesis in Burkholderia Sensu Lato

Different metabolic pathways may supply intermediates for PHA copolymers synthesis. These pathways include fatty acid *β*-oxidation and de novo synthesis (Figure 3), whose metabolic intermediates are converted into R-3HA-CoA for its subsequent polymerization by PHA synthase into poly(3-hydroxybutyryl-*co*-3-hydroxyacyl) (P(3HB-*co*-3HA)) or PHB [121].

Fatty acid *de novo* synthesis pathway yields the intermediate R-3HA-ACP that is transformed by hydroxyacyl-ACP-CoA transacylase (PhaG) or class III ketoacyl-ACP synthase (FabH) into R-3HA-CoA, which is polymerized by PHA synthase [17,122]. All analyzed strains have a FabH enzyme (Figure 5) that probably provides PHA_scl_ precursors according to a multisequence alignment analysis performed in the present review. A conserved phenylalanine residue (F97) found in FabH sequence from *Burkholderia* s.l. strains, similar to F87 in *E. coli* FabH (data not shown), suggests that FabH is specific to short-length *β*-oxidation intermediates (C2-C4) leading to PHA_scl_. as proposed by Nomura et al. [17]. In contrast, in *Mycobacterium tuberculosis* and a *E. coli* mutant strain, a threonine residue replaces phenylalanine in this position, reducing mcl-related substrates (C10-C16) that lead to PHA_mcl-scl_ copolymer production [17,122]. The *phaG* gene was not found in any of the selected strains. Bioinformatic analysis indicated that the *phaG* gene is present in *Burkholderia pseudomallei*, *Burkholderia mallei, Burkholderia oklahomensis, B. gladioli* and *B. glumae* type strains and *T. caryophylli* AS1.2741 [123]. However, in the present review, these were not considered PhaG homologs as they are located next to *rhl* genes of rhamnolipid synthesis. Furthermore, PhaG enzyme has been reported to be highly similar to a β-ketoacyl reductase (RhlG) with identities of 40–45% [124]. On the other hand, the PHA_mcl_ producer *T. caryophylli* AS 1.274 possesses a highly conserved enzyme with the PhaG of *P. putida* (75% identity) that is unrelated to rhamnolipid synthetic genes (Table 1) [125]. PhaG was not found in *T. caryophylli* DSM 50341^T^ nor in *T. symbiotica* JPY-345^T^, therefore the presence of this enzyme is a strain-specific trait in this genus.

The metabolic intermediates of the *β*-oxidation of fatty acids may be funneled into PHA synthesis (Figure 3). The metabolite 2-trans-enoyl-CoA is converted into (R)-3HA-CoA by the addition of H_2_O to the double bond by the R-specific 2-trans-enoyl-CoA hydratase PhaJ or similar enzymes (MaoC, YfcX) [10,126]. In *Pseudomonas aeruginosa*, two *phaJ* genes encode R-specific 2-trans-enoyl-CoA hydratases with different substrate specificity [127]. The *phaJ* gene have been localized downstream of the *phaP* and *phaC* genes [128]. The closely related *P. terrae* DSM 17804^T^, *P. hospita* DSM 17164^T^ and *Paraburkholderia phymatum* STM815 along with *P. sacchari* LMG 19450^T^ and *Paraburkholderia sprentiae* WSM5005^T^ have at least one *phaJ* gene copy located in a *phaC* gene context. While five *Burkholderia* sensu stricto strains and the closely related *P. xenovorans* LB400^T^, *Paraburkholderia aromaticivorans* BN5^T^ *and Paraburkholderia fungorum* ATCC BAA-463^T^ have one copy of a fused gene coding for (R)-specific enoyl-coA hydratase and phosphate acetyl/butyryl transferase enzymes (PhaJ-Pta) (Figure 5). The precursor R-3HA-CoA may also be obtained from the *β*-oxidation metabolic intermediate 3-ketoacyl-CoA that is reduced by 3-ketoacyl reductase (FabG) [121]. FabG have a wide substrate specificity (C4-C12) in *P. aeruginosa* PAO1 [121] and the *fabG* gene is present in all analyzed strains in 1 to 2 copies. Finally, the intermediate (S)-3-hydroxyacyl-CoA can be metabolized by the multifunctional enzyme 3-hydroxyacyl-CoA epimerase (FadB or FadJ) into its (R)-enantiomer, R-3HA-CoA that could be polymerized to PHA_scl_ or PHA_mcl_ [121]. In *E. coli* and *Bacillus subtilis* the multifunctional enzymes FadB and FabJ display 3-hydroxyacyl-CoA dehydrogenase (HADH), 2-trans-enoyl-CoA hydratase (EDH) and epimerase activities [129]. These FadB and FadJ enzymes showed to be the closest relatives for *B. thailandensis* E264^T^, *P. xenovorans* LB400^T^ and *P. caffeinilytica* CF1^T^ (Figure 5). However, for the rest of strains, the closest enzyme is FadN (also found in *Cupriavidus* and *Bacillus* strains) with the HADH and EDH conserved domains, but without epimerase activity evidence [130,131].

**Figure 6:**
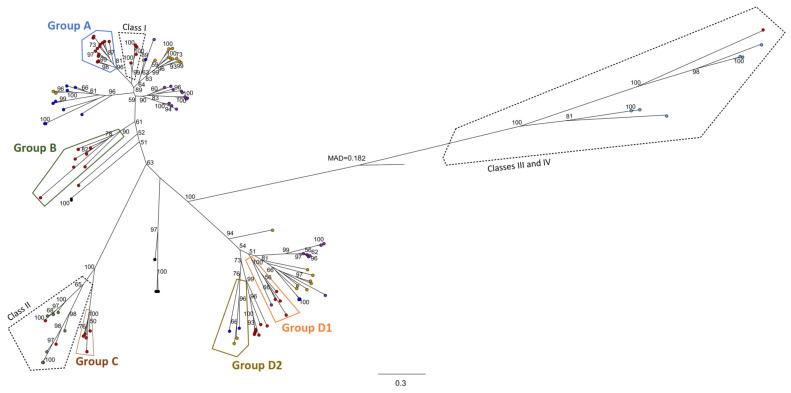
Maximum likelihood phylogenetic tree of PhaC homologues from bacteria of the order *Burkholderiales*. Each amino acidic sequence is represented with a circle. PHA synthases from (•) *Burkholderiaceae*, (•) *Alcaligenaceae*, (•) *Comamonadaceae* and (•) *Oxalobacteraceae*, class II PHA synthases from (•) *Pseudomonas*, class III and IV PHA synthases from (•) *Bacillus*, *Synechocystis*, *Allochromatium*, *Rubrobacter* and *Alcanivorax*. Classes I-IV PHA synthases were included for comparison. Sequences were retrieved by BlastP from IMG database using class I PHA synthase from *C. necator* H16 as the query. Aminoacidic sequences were aligned with a progressive method (MAFFT software) and manually edited. Tree was constructed with Maximum likelihood algorithm (RaXML software), LG+G+I+F substitution model, 1000 bootstrap replicates and rooted using Minimal Ancestral Deviation (MAD) method. Groups A, B, C and D correspond to phylogenetic groups defined in Figure S1.

Valeric acid (VA) can be converted into 3-ketovaleryl-CoA, as an intermediate from *β*-oxidation, or into acetyl-CoA and propionyl-CoA through *β*-oxidation. Propionyl-CoA can be synthesized from propionic acid, pyruvic acid or levulinic acid (LA), fatty acids, threonine, methionine, valine, isoleucine and succinyl-CoA. Propionyl-CoA and acetyl-CoA may be condensed by ketothiolase (BktB) into 3-ketovaleryl-CoA, which is reduced by a ketoreductase (PhaB) into 3-hydroxyvaleryl-CoA, and that finally is polymerized by a PHA synthase into P(3HB-*co*-3HV) [132]. The *bktB* gene has been identified in the majority of *Burkholderia*, *Caballeronia* and *Trinickia* strains with the exception of *P. sacchari* LMG 19450^T^, *P. caffeinilytica* CF1^T^ and *C. sordidicola* LMG 22029. Conversely, only *Burkholderia staibilis* ATCC BAA67^T^, *Burkholderia pyrrocinia* DSM 10685^T^ and *Burkholderia stagnali*s LMG 28156^T^ harbor the *bktB* gene of all analyzed *Burkholderia* sensu stricto strains (Figure 6). Additionally, VA can be obtained from LA, converted by the enzyme acyl-CoA synthetase LvaE to levulinyl-CoA (LA-CoA), and then reduced to 4-hydroxyvaleryl-CoA (4HV-CoA), which is phosphorylated to 4-phosphovaleryl-CoA (4PV-CoA). 4PV-CoA is dephosphorylated to pentenoyl-CoA that is hydrated into 3-hydroxyvaleryl-CoA (3HV-CoA), which can be funneled into PHA biosynthesis or *β*-oxidation [133].

*Burkholderia* s.l. strains showed potential for the synthesis of a wide diversity of PHA_scl_ and PHA_mcl_ precursors. For example, the PHA_scl_-producing strain *B. cepacia* JCM15050 that expresses the *phaC* gene from the PHA_mcl_-producing strain *Aeromonas caviae* synthesizes P(3HB*-co-*3HA) [134]. Hence, PHA synthase substrate specificity along with the precursor availability are key factors that determine the monomeric composition of PHAs.

## 4. PHA Synthases in *Burkholderia* Sensu Lato Strains

PHA synthases are classified into four groups based on the primary structure, the subunit composition, and the substrate specificity [13,135]. Class I and class II PHA synthases are the most common enzymes in bacteria. Class I PHA synthases are homodimers of PhaC that are capable to polymerize mainly PHA monomers of 3–5 carbon chain-length but also polymerize medium chain length monomers (e.g., hydroxyhexanoate) [13,53,135]. The class I PHA synthase of *C. necator* H16 has been widely characterized [13,40,136]. Class II PHA synthases possess one subunit (PhaC1 or PhaC2) and synthesizes PHA_mcl_ from precursors derived mainly from *β*-oxidation or the de novo biosynthesis of fatty acids [13,63,137,138]. Class II PHA synthases have been reported in diverse *Pseudomonas* species. The *phaC* genes encoding PHA synthases are well known mainly in *Cupriavidus* and *Pseudomonas* [64,139] and are well conserved among Betaproteobacteria and Gammaproteobacteria [74]. Class III PHA synthases are heterodimers composed of the subunits PhaC and PhaE and polymerize PHA_scl_ [13,39]. The Proteobacterium *Allochromatium vinosum*, the cyanobacterium *Synechocystis* sp. and archaea such as *Haloarchaea* possess class III enzymes. Class IV PhaC synthases are heterodimers composed of PhaC and PhaR subunits [13,16]. Class IV PHA synthases mostly use short chain-length monomers but can also polymerize other monomers. *Bacillus megaterium* and *Bacillus cereus* class IV PHA synthases have been described [140]. In this review, we observed that class I PHA synthase is the most common type of PHA synthases in *Burkholderia* s.l., whereas class II PHA synthases are present in specific strains. One class III PHA synthase was found in *Caballeronia grimmae* LMG 27580^T^, indicating that this type of synthases is not common among *Burkholderia* s.l.

PHA synthases contain an extended lipase box-like sequence G-G/S-X-C-X-G/A-G in the active site [135,136,137]. The PHA synthases of *Burkholderia* s.l. bacteria contain the lipase-like box sequence G-X-C-X-G-G/A. This lipase box possesses a Cys that is involved in the polymerization process by bounding covalently the substrate, generating the intermediate Cys-S-3HB. The catalytic triad C-H-D, which is crucial for the activity [136], is present in *Burkholderia* s.l. PHA synthases (Figure S2).

The substrate specificity of the PHA synthase influences the monomer composition of the PHA. *B. cepacia* IPT 64 synthesizes from gluconate or sucrose a copolymer composed of PHB (96.5%) and poly(3-hydroxy-4-penteanoate) (P(3H4PE)) (3.5%) [141]. *B. cepacia* IPT64 phaC1 mutant synthesizes both homopolymers but strongly increasing the relative P(3H4PE) concentration (32%), indicating that the wild type strain possesses at least two PHA synthases with different substrate specificity [141]. *B. contaminans* IPT 553, which is able to accumulate PHB, P(3HV) and polyhydroxydecanoate (P(3HDd)) from unrelated carbon sources (i.e., glucose, sucrose glucose/casein and sucrose/casein mixture), has a class I PHA synthase [45]. The diversity of monomers synthesized by strain IPT 553 could be associated to the *phaJ* genes. *T. caryophylli* AS 1.2741 possesses two class II PHA synthases encoded by two *phaC* gene copies separated by the PHA depolymerase encoding *phaZ* gene. The synthesis of PHA_scl_ of 3HB and PHA_mcl_ of 3HD from butyrate, and PHA_mcl_ of 3HHx, 3HO and 3HD from glucose, octanoate and glucose/octanoate by *T. caryophylli* AS 1.2741 is reported [59,125]. *E. coli* KM32B expressing the *phaC1* gene or *phaC2* gene from strain AS 1.2741, produces PHA_mcl_ of 3HHx, 3HO and 3HD from octanoate or decanoate [125]. *P. sacchari* LMG 19450^T^ synthesizes the copolymer P(3HB*-co-*3HHx) with a 3HHx content of 0.14–0.46% mol from glucose and hexanoate [39]. These results suggest that some PHA synthases found in *Burkholderia* s.l. strains, enable them to produce diverse biopolymers with different physicochemical properties, stability and availability [125,137].

To identify the phylogenetic relationships of PHA synthases in *Paraburkholderia*, *Burkholderia*, *Caballeronia*, *Trinickia*, *Mycetohabitans* and *Robbsia* genera, a survey in the available genomic data was performed. Currently, >1000 *Burkholderia*, *Paraburkholderia* and *Caballeronia* genomes are deposited on the comprehensive IMG/M database [142]. For the analyses of the *phaC* genes, we selected all complete genomes available from *Burkholderia* (168), *Paraburkholderia* (16), *Mycetohabitans* (1) and nine draft genomes of *Caballeronia* (6), *Trinickia* (2) and *Robbsia* (1) strains. The genomes of 194 strains of *Burkholderia* s.l. were retrieved from IMG/M database (https://img.jgi.doe.gov/index.html, accessed at 15 January 2021) and NCBI databases (https://www.ncbi.nlm.nih.gov/genome/, accessed at 15 January 2021). Then, the presence of the *phaC* gene was identified in the selected genomes using the BlastP algorithm provided by IMG/M database [142]. The amino acid sequence of PHA synthase from *C. necator* H16 (accession number P23608) was the query for carrying out the search. The proteins that displayed ≥30% amino acid identity, 70% coverage with the *C. necator* H16 PhaC were further studied.

All analyzed genomes possess at least one copy of the *phaC* gene, suggesting that all strains produce PHA. Additionally, 84% of the *Burkholderia* s.l. analyzed strains possess more than one *phaC* gene copy, indicating that *phaC* redundancy is a usual trait.

A preliminary phylogenetic tree delineated four distinctive groups (A, B C, and D) of PhaC amino acid sequences from *Paraburkholderia*, *Burkholderia* and *Caballeronia* genomes (Figure S1). Sequences of PhaC representatives of each group obtained in Figure S1 were selected for a reconstruction of their maximum likelihood phylogeny with the PhaC found in bacteria from the order *Burkholderiales* (Figure 6). This tree was constructed with 112 PhaC sequences from 56 *Burkholderiales* genomes (13 *Burkholderiaceae*, 16 *Alcaligenaceae*, 11 *Comamonadaceae* and 16 *Oxalobacteraceae*). Each genome harbors 1 to 6 copies of PHA synthases. Groups A and B PhaC of the *Burkholderia* s.l. strains are closely related to class I PhaC from *C. necator* H16 (Figure 6). Several of these strains are PHA_scl_ producers [14,40,43,56]. Group C PhaC are closely related to class II PhaC from *Pseudomonas* species. PhaC found in the PHA_mcl_ producing strain *T. caryophylli* AS 1.2741 are found in group C. Therefore, these are interesting candidates to study PHA_mcl_ production as several of these strains that harbor group C synthases also have the metabolic pathways for the supply of PHA_scl_ and PHA_mcl_ precursors (Figure 3 and Figure 5). Interestingly, groups D1 and D2 form a clade that is not related to any described class of PhaC. Strains harboring a group D PhaC produce PHA_scl_ (PHB and P(3HB-*co*-3HV)) and PHA_mcl_ (P(3HB-*co*-3HHx)) [39,44]. A similar finding was reported in recently isolated Janthinobacterium strains, whose PhaC2 showed a distinct clade from the known classes of PHA synthases, proposing a new PHA synthase class [143]. A multiple sequence alignment of PhaCs belonging to the groups identified within *Burkholderiales*, and model PhaC of classes I, II, III and IV was performed in order to describe key amino acidic residues described in previous structural analyses of Wittenborn et al., [136] and Kim et al., [144] (Figure S2). The substrate binding residues I252, L253, T393 and T397 and substrate tunnel residues Y445, I482 and V483 of class I PhaC are highly conserved within groups A and B, while class II and group C PhaC are less conserved in these sites. Conversely, groups D1 and D2 showed to be poorly conserved in relation to classes I‒IV at these sites. These findings show that PHA synthases are more diverse than previously thought and suggests a possible new class of PHA synthases in the analyzed *Burkholderia* s.l. strains and within the *Burkholderiales* order (Figure 6 and Figure S2).

## 5. Gene Synteny of the *phaC* Gene Cluster in *Burkholderia* Sensu Lato

**Figure 7:**
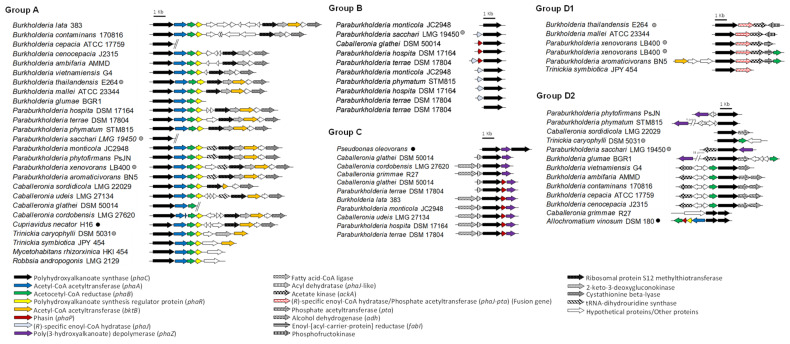
Gene contexts related to *phaC* gene of representative bacteria of the *Burkholderia* sensu lato group. PhaC homologous of *C. necator* H16 and *pha* gene clusters in which the *phaC* gene is present. Gene contexts are arranged according to the neighbor-joining phylogeny of their PhaC amino acidic sequence shown in Figure S1. Branches tagged with black dots indicate sequences with confirmed functionality not belonging to *Burkholderia* s.l. genera that have been incorporated for additional phylogenetic comparisons, while gray dots indicate *Burkholderia* s.l. species with reported polyhydroxyalkanoate synthesis. The *phaC* genes are in black. The function of the genes included in the clusters are indicated at the bottom. The sizes of genes are at a scale depicted in the 1 kb black bar above each group organization.

Gene cluster organization of representative *phaC* genes of the selected *Burkholderia* s.l. identified in Figure S1 are shown in Figure 7 arranged according to their phylogenetic placement (Figure 6 and Figure S1).

Species arranged in group A have the *phaCABR* gene cluster organization with a close relation of PHA synthases from class I (*C. necator* H16). Remarkably, 191 of the 194 reviewed genomes of the preliminary phylogenetic analysis (Figure S1), harbor the *phaCABR* gene cluster, which is the most frequent *pha* gene arrangement. The PHA synthases encoded in the *phaCABR* clusters possess ~60% amino acid identity with *C. necator* H16 class I PHA synthase, which carry the same gene cluster. PHA-producing strains belonging to *P. sacchari* LMG 19450^T^, *P. xenovorans* LB400^T^, *B. thailandensis* E264^T^ and *B. cenocepacia* J2315 possess this gene organization. Interestingly, Hiroe et al. [145] constructed plasmids with different *pha* gene configurations from strain H16 (*phaABC, phaACB, phaBAC phaBCA phaCAB* and *phaCBA*), using *E. coli* DH5α as chassis for biomass and PHB production analysis. Notably, strain DH5α carrying the non-natural *phaCBA* genes arrangement showed the highest PHB production. Nevertheless, the strain that harbor *phaCAB* genes configuration, which is the most typical gene organization in *Burkholderia* s.l. species, displayed the second highest PHB production. This suggests that natural strains have been selected a gene configuration that favors higher PHB and biomass yield, and synthesis of relatively low-molecular-weight polymers [145]. No functional evidence for PHAs synthesis has been described so far in the novel *Caballeronia*, *Mycetohabitans* and *Robbsia* genera. However, all strains reviewed have one *phaC* gene belonging to the group A, strongly suggesting that *Caballeronia*, *Mycetohabitans* and *Robbsia* strains synthesize PHA (Figure 7).

The *phaC* genes that belong to group B are generally in conjunction with one or two additional PHA-related genes such as the *phaP* gene and/or the *phaJ* gene (Figure 7). All the bacteria that harbor a *phaC* gene copy arranged in group B exhibit an additional copy of the *phaC* gene located in the *phaCABR* gene cluster of group A. In *Pseudomonas* and *Bacillus* strains, PhaJ convert *β*-oxidation intermediate 2-trans-enoyl-CoA into to (R)-3-hydroxyacyl-CoA for PHA_mcl_ synthesis [65,138]. The *phaP* gene encodes the surface protein phasin that covers PHA storage granules, playing an important role preventing coalescence of granules and regulation of particle size [146]. Due to the proximity of these genes with a *phaC* gene, it was proposed a possible participation of these genes in PHA synthesis, increasing the PHA diversity produced by these strains.

Remarkably, the *phaC* genes arranged in group C (Figure 7) encode PHA synthases that showed closer similarity with the class II PhaC from *P. putida* (named previously *P. oleovorans*) [19]. In the genomic context of this *phaC* gene are located the *phaZ* gene that encodes the PHA depolymerase, the *phaP* gene and the *maoC* gene. The *maoC* gene encodes a novel enoyl-CoA hydratase, which connects the *β*-oxidation with PHA biosynthetic pathway in a *E. coli fadB* gene mutant defective in fatty acid *β*-oxidation, suggesting that the MaoC enzyme could replace PhaJ [147]. These data suggest that *phaC* gene from group C is involved in PHA biosynthesis.

The analysis of the group D showed that two subgroups could be distinguished (Figure 7). In the group D1, the genomic context of the *phaC* gene possesses an unusual fused gene, which apparently is a fusion between the (R)-specific enoyl-CoA hydratase encoding *phaJ* gene and a phosphate acetyltransferase encoding *pta* gene (Figure 7). Moreover, the *ackA* gene that encodes an acetate kinase is present in this genomic context. The phosphate acetyltransferase (*pta* gene) interconverts acetyl-CoA and acetyl phosphate, whereas the acetate kinase (*ackA* gene) catalyzes the conversion of acetate into acetyl phosphate in the acetate pathway [148]. Remarkably, the overexpression of the *pta* and *ackA* genes in *E. coli* improves acetate assimilation and PHA production [149]. The close presence to the *phaC* gene homologue of genes encoding enzymes of acetate metabolism may have a function related to storage acetate excess per se or acetate-producing carbon sources for PHA synthesis. Furthermore, the gene products of the *phaC*-*pta*-*ack*-*fabI* organization belonging to group D1 (Figure 7) displayed high global identities with the gene products ORF (2–24%), Pta (56–58%), Ack (45–47%), FabI (46–50%) encoded in the ORF-*pta-ack-fabI* gene organization that was found to be co-transcribed and up-regulated during phosphate starvation through *phoB* regulon in *Sinorhizobium meliloti* [150]. The genome context of the *phaC* gene belonging to group D1 suggests that a wide diversity of substrates may be used for PHA synthesis. Interestingly, the only two strains without the canonical *phaCABR* gene organization of the 194 strains analyzed in the preliminary *phaC* gene search (Figure S1), the opportunistic human pathogen *B. pseudomallei* MSHR435 [151] and *B. humptydooensis* strain MSMB1588 isolated from soil in Australia [152], possess the *phaC* gene that belongs to D1 group and the *phaC*/*phaJ*-*pta*/*ackA* gene organization.

Finally, in the group D2, the *phaC* gene is clustered frequently with the *phaB* gene, but also with the *pta* gene and the *ackA* gene as observed in the group D1. Sometimes the *phaZ* gene is also present in this genomic neighborhood. Mendonҁa et al. [39] reported that *P. sacchari* LMG 19450^T^, which have three *phaC* gene copies that belong to groups A, C and D2 (Figure 7), synthesizes short and medium length chain copolymers P(3HB-*co*-3HHx) from glucose and hexanoic acid. This suggests a possible role of the PHA synthases encoded by these *phaC* gene copies in this type of polymer. Additionally, *B. contaminans* 170,816 has a *phaC* gene that belongs to group D2 (Figure 7). However, PHA synthesis by strain 170,816 has not been reported. Nevertheless, *B. contaminans* strains IPT 553 and Kad1, whose genomes have not been sequenced, synthesize PHA_scl_ and PHA_mcl_ [45,58]. Finally, a class III PHA synthase subunit was found next to the *phaE* gene in *C. grimmae* LMG 27580^T^.

Exceptionally, it was found that only one *phaC* gene of the fungal-associated *strain P. terrae* DSM 17804^T^ [153] apparently does not belong to any group described (Figure 5 and Figure S1). This *phaC* copy have an aminoacidic substitution in the well-known C-D-H catalytic triad where an arginine residue replaces the histidine that is found highly conserved in the other PHA synthases (Figure S2). Strain DSM 17804^T^ has a large genome (~10 Mb) that contains six *phaC* gene copies in different genomic contexts. The unusual high *phaC* gene redundancy of *P. terrae* strain DSM 17804^T^ has not been observed in any other *Burkholderia* s.l. strain (Figure 5). One DSM 17804^T^ *phaC* gene copy belongs to the canonical *phaCABR* cluster of the group A, while another four copies are included in groups B and C (Figure 6). In addition, a high *phaC* gene redundancy was observed in the soil strains *Paraburkholderia monticola* JC2948^T^, *P. hospita* DSM 17164^T^ and *C. glathei* LMG 14190^T^, which harbor 4–6 *phaC* gene copies in their genomes [154,155,156].

The *bktB* gene encoding a β-ketothiolase that catalyzes the condensation of acetyl-CoA and propionyl-CoA into 3-hydroxyvalerate (3HV) is present in 135/194 (70%) of *Burkholderia* s.l. strains and is located near the *phaCABR* gene cluster. Exceptionally, the closely related *P. xenovorans* LB400^T^ and *P. aromaticivorans* BN5^T^ have two copies of the *bktB* gene close to the *phaC* gene. One *bktB* gene is located near to the *phaCABR* gene cluster, and the other copy is close to a second *phaC* gene copy that belongs to the group D1 (Figure 7).

The genomic analyses indicate that diverse strains belonging to *Burkholderia* s.l. genera are attractive candidates for the functional study of the biosynthesis of PHAs, including possible new PhaC classes within the clade or new genes and metabolites involved in PHA synthesis.

**Figure 8:**
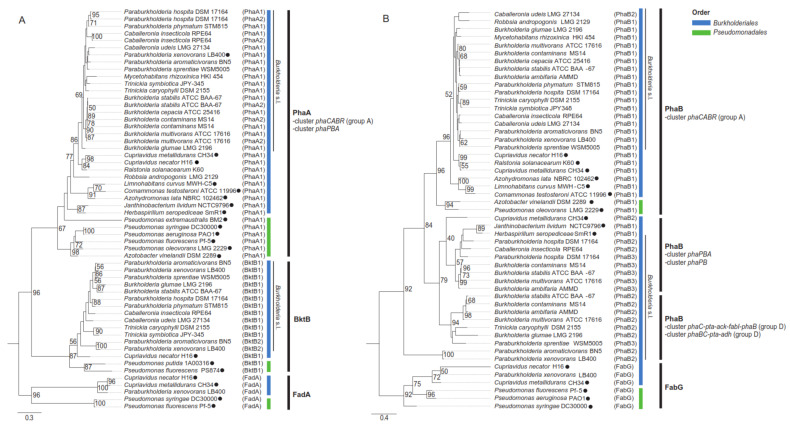
Phylogenetic trees of representative PhaA (**A**) and PhaB (**B**) amino acidic sequences of *Burkholderia* sensu lato (s.l.) strains. The *phaC*-related gene clusters from groups A and D of *Burkholderia* s.l. are shown. Search was carried out through the genomic context of PHA synthases and phasin encoding genes using Refseq and KEGG databases. Organisms of *Burkholderiales* (blue) and *Pseudomonadales* (green) orders, and sequences of BktB, FadA and FabG proteins are included as references (●). Phylogeny was reconstructed using an iterative multiple sequence alignment (Mafft software), maximum-likelihood algorithm with the LG+G+F substitution model with 1000 bootstrap replicates (RaXML software) and rooted according to minimal ancestral deviation method (MAD software).

Most of the PHA genes located in the *phaC* gene context have been proposed before. However, additional *phaA* and *phaB* gene copies located in the *phaPBA* cluster were observed in the *Burkholderia cepacia* complex, *P. hospita* DSM 17164^T^ and *C. glathei* LMG 14190^T^. An additional *phaPB* gene cluster is present in *P. xenovorans* LB400^T^, *P. sprentia*e WSM5005^T^, *P. terrae* DSM 17804^T^, *P. phymatum* STM815^T^ and *C. udeis* LMG 27134^T^. A phylogenetic reconstruction of PhaA and PhaB proteins from the *phaPBA* and *phaPB* gene clusters along with those from groups A and D (Figure 6) was carried out including representative *Burkholderia* s.l. strains, and other *Burkholderiales* and *Pseudomonadales* reference strains (Figure 8).

*Burkholderia* s.l. possess 1–2 gene copies encoding for the PhaA ketothiolases located in two different gene clusters, the *phaCABR* of group A (Figure 7), and the *phaPBA* cluster harboring a phasin-coding gene (*phaP*). These PhaAs amino acid sequences grouped according to the taxonomic relation of these organisms rather than to the cluster type, suggesting a vertical inheritance. Interestingly, *P. xenovorans* LB400 and *P. aromaticivorans* BN5 possess a BktB2 that is clustered in a different branch than the BktB1 clade, closer to *Cupriavidus* and *Pseudomonadales* bacteria (Figure 8A). The PhaB ketoacyl-CoA reductase genes are located in five gene clusters: the *phaCABR* of group A; the *phaBC-pta-ack-adh* of group D1; the *phaC-pta-ack-fabI-phaB* of group D2 (Figure 7); and the *phaP* associated *phaPB* and *phaPBA* gene clusters. In contrast to PhaA, these PhaBs are grouped according to the gene cluster type rather than the strain taxonomic relationship, suggesting a horizontal gene transfer event. The absence of the *phaA* gene in several *pha* gene clusters indicate the relevance of the PhaB presence and diversity, and the loss of the ketothiolase encoding gene or its late entry into the gene clusters [74]. This could be attributed to the physiological role of the PhaB enzyme through NADPH-mediated regulation of PHA metabolism [113]. The PhaP encoded in the *phaPBA* and *phaPB* gene clusters are interesting proteins for studying regulatory and evolutionary issues of the PHA metabolism. The regulation of the PhaP expression has been studied in *C. necator* H16 [157], revealing that transcriptional control is achieved by an autoregulated repressor, which is encoded by the *phaR* gene, having homologues located in the canonical *phaCABR* gene cluster included in group A (Figure 7) and located in nearly all *Burkholderia* s.l. strains (Figure S1). Under cultivation conditions not permissive for PHA biosynthesis in *C. necator* H16, PhaR binds at two sites upstream of the *phaP* gene and represses its transcription [157]. An analysis of the putative regulatory regions [158] upstream of the *phaP* gene in 8 selected *Burkholderia* s.l. strains and *C. necator* H16 allows the identification of a 57 bp-conserved motif (Figure S3). This motif mainly overlaps both PhaR-binding sites in *C. necator* H16, which match the transcriptional start site plus the −10 region and a region immediately upstream of the −35 region of the σ^70^ promoter of the *phaP* gene [157]. The identification of this conserved motif allows to predict that the regulation of PHA biosynthesis in *Burkholderia* s.l. strains would mirror that described in *C. necator* H16, involving the *phaP* promoter and the PhaR transcriptional repressor. Concerning the evolutionary issue, the well conserved canonical *phaCAB* gene cluster allows to infer that these genes were inherited from a recent common ancestor of *Burkholderia* s.l. strains. This is supported by the topology of a phylogenetic tree based in the concatenated amino acidic sequences of PhaC, PhaB and PhaA including the 37 *Burkholderia* s.l. genomes widely used in this review (Figure S4), which shows a strong consistency with the phylogeny of 38 concatenated core genes shown in Figure 1A [159,160,161,162,163]. The only relevant differences among the topologies based in the *phaCAB* genes or core genes are the inclusion of *M. rhizoxinica* HKI 454^T^ in the clade of *Paraburkholderia* species and the exclusion of the phytopathogens *B. plantarii* ATCC 43733^T^ and *B. glumae* LMG 2196^T^ from the *Burkholderia* clade (Figure S4). In any case, the strong conservation of the *phaCAB* gene cluster among *Burkholderia* s.l. species reveals the relevance of PHA biosynthesis for the fitness of this metabolically versatile proteobacteria in different ecological niches regardless of the specific lifestyle of each member.

## 6. Conclusions

*Burkholderia* sensu lato strains synthesize PHA homopolymer and copolymers from different sugars and fatty acids. In this review, the reconstruction of the metabolic pathways of 37 type and representative strains from the *Burkholderi*a sensu stricto, *Paraburkholderia*, *Caballeronia*, *Mycetohabitans*, *Trinickia* and *Robbsia* genera involved in the conversion of sugars and fatty acids into PHAs was performed based on their genome analyses and previous reports. These strains possess the genes to metabolize sugars and fatty acids and related substrates into PHA_scl_ homopolymer and PHA_scl_ or PHA_scl-mcl_ copolymers. In *Burkholderia* s.l. strains, the ED and PP pathways but not the EMP pathway are essential routes for the conversion of sugars and related compounds into PHAs. The *β*-oxidation of fatty acids and fatty acid de novo synthesis are linked with the synthesis of PHAs in *Burkholderia* s.l. strains. *Paraburkholderia* and *Caballeronia* strains exhibited overall higher gene redundancy in carbohydrate and fatty acid metabolism than the rest of *Burkholderia* s.l. strains. The analysis of 194 *Burkholderia* s.l. genomes revealed that all these strains have the *phaC* gene, generally, in two or more copies. The PHA synthases of *Burkholderia* s.l. strains belong to the PHA synthases of class I, II, III and an outliner, and were classified into four phylogenetic groups. Four main *pha* gene organizations were observed in *Burkholderia* s.l. strains. Finally, this review describes genetic determinants related to environmental stress resistance that could be linked to PHA synthesis in *Burkholderia* s.l. genera. The genome analyses indicate that diverse *Burkholderia* s.l. strains are attractive candidates to study the synthesis of diverse PHAs.

## Figures and Tables

**Table 2:** Genome characteristics of 37 *Burkholderia* sensu lato type and representative strains.

Strain	Accession Number	Chr	Plasmids	Size (Mbp)	Contigs	CDS	G+C Content (mol%)
*Burkholderia cepacia* ATCC 25416^T^	GCA_006094315	3	2	8574	5	7619	66.59
*Burkholderia contaminans* MS14	GCA_001029145	3	0	8509	3	7494	66.38
*Burkholderia cenocepacia* J2315^T^	GCA_902830575	-	-	7911	89	7105	66.99
*Burkholderia stabilis* ATCC BAA-67^T^	GCA_001742165	3	0	8528	3	7552	66.42
*Burkholderia pyrrocinia* DSM 10685^T^	GCA_001028665	3	1	7961	4	6920	66.46
*Burkholderia vietnamiensis* LMG 10929^T^	GCA_902830295	-	-	6876	65	5397	66.89
*Burkholderia ambifaria* AMMD^T^	GCA_000959545	3	1	7528	4	6548	66.77
*Burkholderia stagnalis* LMG 28156^T^	GCA_902830275	-	-	8032	149	7039	67.23
*Burkholderia multivorans* ATCC 17616	GCA_000010545	3	1	7009	4	6262	66.69
*Burkholderia thailandensis* E264^T^	GCA_000012365	2	0	6724	2	5656	67.73
*Burkholderia mallei* ATCC 23344^T^	GCA_000011705	2	0	5836	2	4820	68.49
*Burkholderia glumae* LMG 2196^T^	GCA_902832765	-	-	6662	142	5623	68.34
*Burkholderia plantarii* ATCC 43733^T^	GCA_001411805	2	1	8081	3	6715	68.55
*Paraburkholderia graminis* PHS1	GCA_003330785	2	1	7508	3	6510	62.83
*Paraburkholderia caledonica* PHRS4	GCA_003330745	2	1	7305	3	6042	61.93
*Paraburkholderia aromaticivorans* BN5^T^	GCA_002278075	2	6	8908	8	7753	62.94
*Paraburkholderia xenovorans* LB400^T^	GCA_000756045	2	1	9703	3	8321	62.63
*Paraburkholderia phytofirmans* PsJN^T^	GCA_000020125	2	1	8215	3	7175	62.29
*Paraburkholderia fungorum* ATCC BAA-463^T^	GCA_000961515	3	1	9059	4	7898	61.75
*Paraburkholderia caffeinilytica* CF1^T^	GCA_003368325	2	1	8324	3	7142	62.21
*Paraburkholderia sprentiae* WSM5005^T^	GCA_001865575	2	3	7829	5	6699	63.21
*Paraburkholderia megapolitana* LMG 23650^T^	GCA_900113825	-	-	7607	32	6571	62.07
*Paraburkholderia terrae* DSM 17804^T^	GCA_002902925	4	0	10,062	4	8754	61.92
*Paraburkholderia hospita* DSM 17164^T^	GCA_002902965	5	1	11,528	6	9975	61.79
*Paraburkholderia phymatum* STM 815^T^	GCA_000020045	2	2	8676	4	7405	62.29
*Paraburkholderia sacchari* LMG 19450^T^	GCA_000785435 *	-	-	7318	21	6341	64.01
*Paraburkholderia tropica* LMG 22274^T^	GCA_902833865	-	-	8598	53	7619	64.77
*Caballeronia sordidicola* LMG 22029	GCA_001544455 *	-	-	6874	72	6002	60.15
*Caballeronia udeis* LMG 27134^T^	GCA_001544555 *	-	-	10,052	242	8774	60.04
*Caballeronia glathei* LMG 14190^T^	GCA_902833485	-	-	8637	356	7660	64.41
*Caballeronia insecticola* RPE64^T^	GCA_000402035	3	2	6964	5	6266	63.15
*Caballeronia cordobensis* LMG 27620^T^	GCA_001544575 *	-	-	8208	74	7428	63.69
*Caballeronia grimmiae* LMG 27580^T^	GCA_000698555	-	-	6704	160	6024	63.02
*Trinickia caryophylli* DSM 50341^T^	GCA_002879875	-	-	6581	158	5626	64.62
*Trinickia symbiotica* JPY-345^T^	GCA_002934455	-	-	6714	62	5823	63.00
*Mycetohabitans rhizoxinica* HKI 454^T^	GCA_000198775	1	2	3750	3	2875	60.71
*Robbsia andropogonis* LMG 2129^T^	GCA_902833845	-	-	6.33	77	5183	58.86

Chr, number of chromosomes; CDS, coding sequences; type strains are marked with a T superscript; (*) a second version of the genome.

## Data Availability

Genomic data supporting this review is found in Kyoto Encyclopedia of Genes and Genomes (KEGG) (https://www.genome.jp/kegg/, accessed at 15 January 2021), Integrated Microbial Genomes and Metagenomes (IMG/M) database (https://img.jgi.doe.gov/index.html, accessed at 15 January 2021), NCBI databases (https://www.ncbi.nlm.nih.gov/genome/, accessed at 15 January 2021) and Metacyc (https://metacyc.org/, accessed at 15 January 2021) database.

## Supplementary Materials

The following are available online at https://www.mdpi.com/article/10.3390/microorganisms9061290/s1. Table S1. Assimilation of substrates commonly used for PHA production in 37 *Burkholderia* sensu lato type and representative strains. Figure S1: Phylogenetic tree for PhaC of representative *Burkholderia* sensu lato species, Figure S2: Multiple sequence alignment of PHA synthases of four phylogenetic groups in *Burkholderia* sensu lato genomes, Figure S3: Conserved motif in the putative promoter sequence of the *phaP* gene in 8 selected *Burkholderia* sensu lato strains and the model PHA-producer *C. necator* H16 Figure S4: Evolutionary relationships among concatenated PhaA, PhaB and PhaC protein homologues encoded by the conserved canonical *phaCAB* gene cluster of the 37 *Burkholderia* sensu lato genomes.

## References

[1] Geyer R., Jambeck J., Law K.L. (2017). Production, use, and fate of all plastics ever made. Sci. Adv..

[2] Plastics Europe An Analysis of European Plastics Production, Demand and Waste Data. In Plastics-The Facts 2019. https:/www.plasticseurope.org/es/resources/publications/1804-plastics-facts-2019.

[3] Koller M. (2017). Production of Polyhydroxyalkanoate (PHA) Biopolyesters by Extremophiles?. MOJ Polym. Sci..

[4] Sanhueza C., Acevedo F., Rocha S., Villegas P., Seeger M., Navia R. (2019). Polyhydroxyalkanoates as biomaterial for electrospun scaffolds. Int. J. Biol. Macromol..

[5] Sanhueza C., Diaz-Rodriguez P., Villegas P., González Á., Seeger M., Suárez-González J., Concheiro A., Alvarez-Lorenzo C., Acevedo F. (2020). Influence of the carbon source on the properties of poly-(3)-hydroxybutyrate produced by *Paraburkholderia xenovorans* LB400 and its electrospun fibers. Int. J. Biol. Macromol..

[6] Vilchez A., Acevedo F., Cea M., Seeger M., Navia R. (2020). Applications of Electrospun Nanofibers with Antioxidant Properties: A Review. Nanomaterials.

[7] Shahzad K., Ismail I.M.I., Ali N., Rashid M.I., Summan A.S.A., Kabli M.R., Narodoslawsky M., Koller M. (2020). LCA, Sustainability and Techno-Economic Studies for PHA Production. The Handbook of Polyhydroxyalkanoates.

[8] Silva L.F., Taciro M.K., Ramos M.E.M., Carter J.M., Pradella J.G.C., Gomez J.G.C. (2004). Poly-3-hydroxybutyrate (P3HB) production by bacteria from xylose, glucose and sugarcane bagasse hydrolysate. J. Ind. Microbiol. Biotechnol..

[9] Nikel P.I., Pettinari M., Ramírez M., Galvagno M.A., Méndez B.S. (2008). *Escherichia coli* arcA mutants: Metabolic profile characterization of microaerobic cultures using glycerol as a carbon source. J. Mol. Microbiol. Biotechnol..

[10] Lu J., Tappel R.C., Nomura C.T. (2009). Mini-Review: Biosynthesis of poly(hydroxyalkanoates). Polym. Rev..

[11] Ciesielski S., Mozejko-Ciesielska J., Pisutpaisal N. (2015). Plant oils as promising substrates for polyhydroxyalkanoates production. J. Clean. Prod..

[12] Możejko-Ciesielska J., Kiewisz R. (2016). Bacterial polyhydroxyalkanoates: Still fabulous?. Microbiol. Res..

[13] Zou H., Shi M., Zhang T., Xian M., Li L. (2017). Natural and engineered polyhydroxyalkanoate (PHA) synthase: Key enzyme in biopolyester production. Appl. Microbiol. Biotechnol..

[14] Li M., Eskridge K.M., Wilkins M.R. (2019). Optimization of polyhydroxybutyrate production by experimental design of combined ternary mixture (glucose, xylose and arabinose) and process variables (sugar concentration, molar C:N ratio). Bioprocess Biosyst. Eng..

[15] Raza Z.A., Abid S., Banat I.M. (2018). Polyhydroxyalkanoates: Characteristics, production, recent developments and applications. Int. Biodeterior. Biodegrad..

[16] Tsuge T., Fukui T., Matsusaki H., Taguchi S., Kobayashi G., Ishizaki A., Doi Y. (2000). Molecular cloning of two (R)-specific enoyl-CoA hydratase genes from *Pseudomonas aeruginosa* and their use for polyhydroxyalkanoate synthesis. FEMS Microbiol. Lett..

[17] Nomura C.T., Taguchi K., Taguchi S., Doi Y. (2004). Coexpression of genetically engineered 3-ketoacyl-ACP synthase III (fabH) and polyhydroxyalkanoate synthase (phaC) genes leads to short-chain-length-medium-chain-length polyhydroxyalkanoate copolymer production from glucose in *Escherichia coli* JM109. Appl. Environ. Microbiol..

[18] Wang Q., Nomura C.T. (2010). Monitoring differences in gene expression levels and polyhydroxyalkanoate (PHA) production in *Pseudomonas putida* KT2440 grown on different carbon sources. J. Biosci. Bioeng..

[19] Madison L.L., Huisman G.W. (1999). Metabolic engineering of poly(3-hydroxyalkanoates): From DNA to plastic. Microbiol. Mol. Biol. Rev..

[20] Qi Q., Rehm B.H.A. (2001). Polyhydroxybutyrate biosynthesis in *Caulobacter crescentus*: Molecular characterization of the polyhydroxybutyrate synthase. Microbiology.

[21] Park J.M., Kim T.Y., Lee S.Y. (2011). Genome-scale reconstruction and in silico analysis of the *Ralstonia eutropha* H16 for polyhydroxyalkanoate synthesis, lithoautotrophic growth, and 2-methyl citric acid production. BMC Syst. Biol..

[22] Grousseau E., Blanchet E., Déléris S., Albuquerque M.G., Paul E., Uribelarrea J.-L. (2013). Impact of sustaining a controlled residual growth on polyhydroxybutyrate yield and production kinetics in *Cupriavidus necator*. Bioresour. Technol..

[23] Meng D.-C., Shen R., Yao H., Chen J.-C., Wu Q., Chen G.-Q. (2014). Engineering the diversity of polyesters. Curr. Opin. Biotechnol..

[24] Sawana A., Eadeolu M., Gupta R.S. (2014). Molecular signatures and phylogenomic analysis of the genus *Burkholderia*: Proposal for division of this genus into the emended genus *Burkholderia* containing pathogenic organisms and a new genus *Paraburkholderia* gen. nov. harboring environmental species. Front. Genet..

[25] Dobritsa A.P., Samadpour M. (2016). Transfer of eleven species of the genus *Burkholderia* to the genus *Paraburkholderia* and proposal of *Caballeronia* gen. nov. to accommodate twelve species of the genera *Burkholderia* and *Paraburkholderia*. Int. J. Syst. Evol. Microbiol..

[26] Dobritsa A.P., Samadpour M. (2019). Reclassification of *Burkholderia insecticola* as *Caballeronia insecticola* comb. nov. and reliability of conserved signature indels as molecular synapomorphies. Int. J. Syst. Evol. Microbiol..

[27] Lin Q.-H., Lv Y.-Y., Gao Z.-H., Qiu L.-H. (2020). *Pararobbsia silviterrae* gen. nov., sp. nov., isolated from forest soil and reclassification of *Burkholderia alpina* as *Pararobbsia alpina* comb. nov. Int. J. Syst. Evol. Microbiol..

[28] Chain P.S.G., Denef V.J., Konstantinidis K.T., Vergez L.M., Agulló L., Reyes V.L., Hauser L., Córdova M., Gómez L., González M. (2006). *Burkholderia xenovorans* LB400 harbors a multi-replicon, 9.73-Mbp genome shaped for versatility. Proc. Natl. Acad. Sci. USA.

[29] Pérez-Pantoja D., Donoso R., Agulló L., Córdova M., Seeger M., Pieper D.H., González B. (2012). Genomic analysis of the potential for aromatic compounds biodegradation in *Burkholderiales*. Environ. Microbiol..

[30] Peeters C., Meier-Kolthoff J.P., Verheyde B., De Brandt E., Cooper V.S., Vandamme P. (2016). Phylogenomic study of *Burkholderia glathei*-like organisms, proposal of 13 novel *Burkholderia* Species and emended descriptions of *Burkholderia sordidicola*, *Burkholderia zhejiangensis*, and *Burkholderia grimmiae*. Front. Microbiol..

[31] Agulló L., Romero-Silva M.J., Domenech M., Seeger M. (2017). p-Cymene promotes its catabolism through the p-cymene and the p-cumate pathways, activates a stress response and reduces the biofilm formation in *Burkholderia xenovorans* LB400. PLoS ONE.

[32] Seeger M., Zielinski M., Timmis K.N., Hofer B. (1999). Regiospecificity of dioxygenation of di- to pentachlorobiphenyls and their degradation to chlorobenzoates by the bph-encoded catabolic pathway of *Burkholderia* sp. strain LB400. Appl. Environ. Microbiol..

[33] Seeger M., González M., Cámara B., Muñoz L., Ponce E., Mejías L., Mascayano C., Vásquez Y., Sepúlveda-Boza S. (2003). Biotransformation of natural and synthetic isoflavonoids by two recombinant microbial enzymes. Appl. Environ. Microbiol..

[34] Chirino B., Strahsburger E., Agulló L., González M., Seeger M. (2013). Genomic and functional analyses of the 2-aminophenol catabolic pathway and partial conversion of its substrate into picolinic acid in *Burkholderia xenovorans* LB400. PLoS ONE.

[35] Fuentes S., Méndez V., Aguila P., Seeger M. (2014). Bioremediation of petroleum hydrocarbons: Catabolic genes, microbial communities, and applications. Appl. Microbiol. Biotechnol..

[36] Vargas-Straube M.J., Cámara B., Tello M., Silva F.M., Cárdenas F., Seeger M. (2016). Genetic and functional analysis of the biosynthesis of a non-ribosomal peptide siderophore in *Burkholderia xenovorans* LB400. PLoS ONE.

[37] Vega-Celedón P., Canchignia H., González M., Seeger M. (2016). Biosynthesis of indole-3-acetic acid and plant growth promoting by bacteria. Cultiv. Trop..

[38] Donoso R., Leiva-Novoa P., Zúñiga A., Timmermann T., Recabarren-Gajardo G., González B. (2017). Biochemical and genetic bases of indole-3-acetic acid (auxin phytohormone) degradation by the plant-growth-promoting rhizobacterium *Paraburkholderia phytofirmans* PsJN. Appl. Environ. Microbiol..

[39] Mendonça T.T., Tavares R.R., Cespedes L.G., Rodriguez R.J.S., Schripsema J., Taciro M.K., Gomez J.G., Silva L.F. (2017). Combining molecular and bioprocess techniques to produce poly(3-hydroxybutyrate- co -3-hydroxyhexanoate) with controlled monomer composition by *Burkholderia sacchari*. Int. J. Biol. Macromol..

[40] Urtuvia V., Villegas P., Fuentes S., González M., Seeger M. (2018). *Burkholderia xenovorans* LB400 possesses a functional polyhydroxyalkanoate anabolic pathway encoded by the *pha* genes and synthesizes poly(3-hydroxybutyrate) under nitrogen-limiting conditions. Int. Microbiol..

[41] Lopes M.S.G., Rocha R.C.S., Zanotto S.P., Gomez J.G.C., Da Silva L.F. (2009). Screening of bacteria to produce polyhydroxyalkanoates from xylose. World J. Microbiol. Biotechnol..

[42] Pan W., Perrotta J.A., Stipanovic A.J., Nomura C.T., Nakas J.P. (2012). Production of polyhydroxyalkanoates by *Burkholderia cepacia* ATCC 17759 using a detoxified sugar maple hemicellulosic hydrolysate. J. Ind. Microbiol. Biotechnol..

[43] Kourmentza C., Costa J., Azevedo Z., Servin C., Grandfils C., Freitas V., Reis M. (2018). *Burkholderia thailandensis* as a microbial cell factory for the bioconversion of used cooking oil to polyhydroxyalkanoates and rhamnolipids. Bioresour. Technol..

[44] Mendonça T., Gomez J., Buffoni E., Rodriguez R.J.S., Schripsema J., Lopes M., Silva L. (2014). Exploring the potential of *Burkholderia sacchari* to produce polyhydroxyalkanoates. J. Appl. Microbiol..

[45] Nahar S., Jeong M.-H., Hur J.-S. (2019). Lichen-Associated bacterium, a novel bioresource of polyhydroxyalkanoate (pha) production and simultaneous degradation of naphthalene and anthracene. J. Microbiol. Biotechnol..

[46] Timm A., Steinbüchel A. (1990). Formation of polyesters consisting of medium-chain-length 3-hydroxyalkanoic acids from gluconate by *Pseudomonas aeruginosa* and other fluorescent *pseudomonads*. Appl. Environ. Microbiol..

[47] Cesário M.T., Raposo R.S., de Almeida M.C.M., van Keulen F., Ferreira B., da Fonseca M.M. (2014). Enhanced bioproduction of poly-3-hydroxybutyrate from wheat straw lignocellulosic hydrolysates. New Biotechnol..

[48] Lopes M.S., Gomez J.G., Silva L.F. (2009). Cloning and overexpression of the xylose isomerase gene from *Burkholderia sacchari* and production of polyhydroxybutyrate from xylose. Can. J. Microbiol..

[49] Dietrich K., Dumont M.-J., Schwinghamer T., Orsat V., Del Rio L.F. (2018). Model study to assess softwood hemicellulose hydrolysates as the carbon source for PHB production in *Paraburkholderia sacchari* IPT 101. Biomacromolecules.

[50] Gomez J.G.C., Rodrigues M.F.A., Alli R.C.P., Torres B.B., Netto C.L.B., Oliveira M.S., Da Silva L.F. (1996). Evaluation of soil gram-negative bacteria yielding polyhydroxyalkanoic acids from carbohydrates and propionic acid. Appl. Microbiol. Biotechnol..

[51] Cesário M.T., Raposo R.S., De Almeida M.C.M., Van Keulen F., Ferreira B., Telo J., da Fonseca M.M. (2014). Production of poly(3-hydroxybutyrate-co-4-hydroxybutyrate) by *Burkholderia sacchari* using wheat straw hydrolysates and gamma-butyrolactone. Int. J. Biol. Macromol..

[52] Raposo R.S., De Almeida M.C.M., Da Fonseca M., Cesário M.T. (2017). Feeding strategies for tuning poly (3-hydroxybutyrate-co-4-hydroxybutyrate) monomeric composition and productivity using *Burkholderia sacchari*. Int. J. Biol. Macromol..

[53] Urtuvia V., Villegas P., González M., Seeger M. (2014). Bacterial production of the biodegradable plastics polyhydroxyalkanoates. Int. J. Biol. Macromol..

[54] Acevedo F., Villegas P., Urtuvia V., Hermosilla J., Navia R., Seeger M. (2018). Bacterial polyhydroxybutyrate for electrospun fiber production. Int. J. Biol. Macromol..

[55] Ramsay J.A., Hassan M.-C.A., Ramsay B.A. (1995). Hemicellulose as a potential substrate for production of poly (β-hydroxyalkanoates). Can. J. Microbiol..

[56] Ramsay B.A., Ramsay J.A., Cooper D.G. (1989). Production of poly-β-hydroxyalkanoic acid by *Pseudomonas cepacia*. Appl. Environ. Microbiol..

[57] Keenan T., Tanenbaum S., Stipanovic A., Nakas J. (2004). Production and characterization of poly-β-hydroxyalkanoate copolymers from *Burkholderia cepacia* utilizing xylose and levulinic acid. Biotechnol. Prog..

[58] Al-Kaddo K.B., Mohamad F., Murugan P., Tan J.S., Sudesh K., Samian M.R. (2020). Production of P(3HB-co-4HB) copolymer with high 4HB molar fraction by *Burkholderia contaminans* Kad1 PHA synthase. Biochem. Eng. J..

[59] Hang X., Zhang G., Wang G., Zhao X., Chen G.-Q. (2002). PCR cloning of polyhydroxyalkanoate biosynthesis genes from *Burkholderia caryophylli* and their functional expression in recombinant *Escherichia coli*. FEMS Microbiol. Lett..

[60] Habe H., Sato S., Morita T., Fukuoka T., Kirimura K., Kitamoto D. (2015). Bacterial production of short-chain organic acids and trehalose from levulinic acid: A potential cellulose-derived building block as a feedstock for microbial production. Bioresour. Technol..

[61] Ashby R.D., Solaiman D.K., Nuñez A., Strahan G.D., Johnston D.B. (2018). *Burkholderia sacchari* DSM 17165: A source of compositionally-tunable block-copolymeric short-chain poly(hydroxyalkanoates) from xylose and levulinic acid. Bioresour. Technol..

[62] Volova T.G., Vinogradova O.N., Zhila N.O., Kiselev E.G., Peterson I.V., Vasilev A.D., Sukovatyi A.G., Shishatskaya E. (2017). Physicochemical properties of multicomponent polyhydroxyalkanoates: Novel aspects. Polym. Sci. Ser. A.

[63] Jiang X., Luo X., Zhou N.-Y. (2015). Two polyhydroxyalkanoate synthases from distinct classes from the aromatic degrader *Cupriavidus pinatubonensis* JMP134 exhibit the same substrate preference. PLoS ONE.

[64] Prieto A., Escapa I.F., Martinez V., Dinjaski N., Herencias C., De La Peña F., Tarazona N., Revelles O. (2016). A holistic view of polyhydroxyalkanoate metabolism in *Pseudomonas putida*. Environ. Microbiol..

[65] Kihara T., Hiroe A., Ishii-Hyakutake M., Mizuno K., Tsuge T. (2017). *Bacillus cereus*-type polyhydroxyalkanoate biosynthetic gene cluster contains R-specific enoyl-CoA hydratase gene. Biosci. Biotechnol. Biochem..

[66] Álvarez-Santullano N., Villegas P., Sepúlveda M., Vilchez A., Donoso R., Pérez-Pantoja D., Navia R., Acevedo F., Seeger M., Koller M. (2020). Production of polyhydroxyalkanoates by *Paraburkholderia* and *Burkholderia* species: A journey from the genes through metabolic routes. The Handbook of Polyhydroxyalkanoates.

[67] Parte A.C., Carbasse J.S., Meier-Kolthoff J.P., Reimer L.C., Göker M. (2020). List of Prokaryotic names with standing in nomenclature (lpsn) moves to the DSMZ. Int. J. Syst. Evol. Microbiol..

[68] Mendler K., Chen H., Parks D.H., Lobb B., Hug L., Doxey A.C. (2019). AnnoTree: Visualization and exploration of a functionally annotated microbial tree of life. Nucleic Acids Res..

[69] Parks D.H., Chuvochina M., Waite D.W., Rinke C., Skarshewski A., Chaumeil P.A., Hugenholtz P. (2018). A standardized bacterial taxonomy based on genome phylogeny substantially revises the tree of life. Nat. Biotechnol..

[70] Caspi R., Billington R., A Fulcher C., Keseler I.M., Kothari A., Krummenacker M., Latendresse M., E Midford P., Ong Q., Ong W.K. (2018). The MetaCyc database of metabolic pathways and enzymes. Nucleic Acids Res..

[71] Segata N., Börnigen D., Morgan X.C., Huttenhower C. (2013). PhyloPhlAn is a new method for improved phylogenetic and taxonomic placement of microbes. Nat. Commun..

[72] Pratama A.A., Jiménez D.J., Chen Q., Bunk B., Spröer C., Overmann J., Van Elsas J.D. (2020). Delineation of a subgroup of the genus *Paraburkholderia*, including *P. terrae* DSM 17804T, *P. hospita* DSM 17164T, and four soil-isolated fungiphiles, reveals remarkable genomic and ecological features—proposal for the definition of a *P. hospita* species cluster. Genome Biol. Evol..

[73] Feng Y., Cronan J.E. (2009). *Escherichia coli* unsaturated fatty acid synthesis. J. Biol. Chem..

[74] Kutralam-Muniasamy G., Marsch R., Pérez-Guevara F. (2018). Investigation on the evolutionary relation of diverse polyhydroxyalkanoate gene clusters in betaproteobacteria. J. Mol. Evol..

[75] Urakami T., Ito-Yoshida C., Araki H., Kijima T., Suzuki K.-I., Komagata K. (1994). Transfer of Pseudomonas plantarii and *Pseudomonas glumae* to Burkholderia as *Burkholderia* spp. and description of *Burkholderia vandii* sp. nov. Int. J. Syst. Bacteriol..

[76] Vandamme P., Holmes B., Vancanneyt M., Coenye T., Hoste B., Coopman R., Revets H., Lauwers S., Gillis M., Kersters K. (1997). Occurrence of multiple genomovars of *Burkholderia cepacia* in cystic fibrosis patients and proposal of *Burkholderia multivorans* sp. nov. Int. J. Syst. Bacteriol..

[77] Seth-Smith H.M., Casanova C., Sommerstein R., Meinel D.M., Abdelbary M.M., Blanc D.S., Droz S., Führer U., Lienhard R., Lang C. (2019). Phenotypic and genomic analyses of *Burkholderia stabilis* clinical contamination, Switzerland. Emerg. Infect. Dis..

[78] Brämer C., Vandamme P., Da Silva L.F., Gomez J.G.C., Steinbüchel A. (2001). Polyhydroxyalkanoate-accumulating bacterium isolated from soil of a sugar-cane plantation in Brazil. Int. J. Syst. Evol. Microbiol..

[79] Gillis M., Van Van T., Bardin R., Goor M., Hebbar P., Willems A., Segers P., Kersters K., Heulin T., Fernandez-Fernandez M.P. (1995). Polyphasic taxonomy in the genus *Burkholderia* Leading to an emended description of the genus and proposition of *Burkholderia vietnamiensis* sp. nov. for n2-fixing isolates from rice in Vietnam. Int. J. Syst. Bacteriol..

[80] Coenye T., Laevens S., Willems A., Olén M., Hannat W., Govan J., Gillis M., Falsen E., Vandamme P. (2001). *Burkholderia fungorum* sp. nov. and *Burkholderia caledonica* sp. nov., two new species isolated from the environment, animals and human clinical samples. Int. J. Syst. Evol. Microbiol..

[81] De Smet B., Mayo M., Peeters C., Zlosnik J., Spilker T., Hird T.J., Lipuma J.J., Kidd T., Kaestli M., Ginther J.L. (2015). *Burkholderia stagnalis* sp. nov. and *Burkholderia territorii* sp. nov., two novel *Burkholderia cepacia* complex species from environmental and human sources. Int. J. Syst. Evol. Microbiol..

[82] Vandamme P., Opelt K., Knöchel N., Berg C., Schönmann S., De Brandt E., Eberl L., Falsen E., Berg G. (2007). *Burkholderia bryophila* sp. nov. and *Burkholderia megapolitana* sp. nov., moss-associated species with antifungal and plant-growth-promoting properties. Int. J. Syst. Evol. Microbiol..

[83] Brett P.J., DeShazer D., Woods D.E. (1998). Note: *Burkholderia thailandensis* sp. nov., a *Burkholderia pseudomallei*-like species. Int. J. Syst. Bacteriol..

[84] Victor I.U., Kwiencien M., Tripathi L., Cobice D., McClean S., Marchant R., Banat I.M. (2019). Quorum sensing as a potential target for increased production of rhamnolipid biosurfactant in *Burkholderia thailandensis* E264. Appl. Microbiol. Biotechnol..

[85] Yabuuchi E., Kosako Y., Oyaizu H., Yano I., Hotta H., Hashimoto Y., Ezaki T., Arakawa M. (1992). Proposal of *Burkholderia* gen. nov. and transfer of seven species of the genus *Pseudomonas* homology group ii to the new genus, with the type species *Burkholderia cepacia* (Palleroni and Holmes 1981) comb. nov. Microbiol. Immunol..

[86] Lee Y., Jeon C.O. (2018). *Paraburkholderia aromaticivoran*s sp. nov., an aromatic hydrocarbon-degrading bacterium, isolated from gasoline-contaminated soil. Int. J. Syst. Evol. Microbiol..

[87] Barberan A. (2016). International Journal of Systematic and Evolutionary Microbiology (IJSEM) Phenotypic Database. Figshare Dataset. https://figshare.com/articles/dataset/International_Journal_of_Systematic_and_Evolutionary_Microbiology_IJSEM_phenotypic_database/4272392.

[88] Sessitsch A., Coenye T., Sturz A.V., Vandamme P., Barka E.A., Salles J.F., Van Elsas J.D., Faure D., Reiter B., Glick B.R. (2005). *Burkholderia phytofirmans* sp. nov., a novel plant-associated bacterium with plant-beneficial properties. Int. J. Syst. Evol. Microbiol..

[89] Gao Z., Yuan Y., Xu L., Liu R., Chen M., Zhang C. (2016). *Paraburkholderia caffeinilytica* sp. nov., isolated from the soil of a tea plantation. Int. J. Syst. Evol. Microbiol..

[90] Yang H.-C., Im W.-T., Kim K.K., An D.-S., Lee S.-T. (2006). *Burkholderia terrae* sp. nov., isolated from a forest soil. Int. J. Syst. Evol. Microbiol..

[91] Vandamme P., De Brandt E., Houf K., Salles J.F., Van Elsas J.D., Spilker T., LiPuma J.J. (2013). *Burkholderia humi* sp. nov., *Burkholderia choica* sp. nov., *Burkholderia telluris* sp. nov., *Burkholderia terrestris* sp. nov. and *Burkholderia udeis* sp. nov.: *Burkholderia glathei*-like bacteria from soil and rhizosphere soil. Int. J. Syst. Evol. Microbiol..

[92] Takeshita K., Tamaki H., Ohbayashi T., Meng X.-Y., Sone T., Mitani Y., Peeters C., Kikuchi Y., Vandamme P. (2018). *Burkholderia insecticola* sp. nov., a gut symbiotic bacterium of the bean bug *Riptortus pedestris*. Int. J. Syst. Evol. Microbiol..

[93] Draghi W.O., Peeters C., Cnockaert M., Snauwaert C., Wall L.G., Zorreguieta A., Vandamme P. (2014). *Burkholderia cordobensis* sp. nov., from agricultural soils. Int. J. Syst. Evol. Microbiol..

[94] Santos P.E.-D.L., Palmer M., Chávez-Ramírez B., Beukes C., Steenkamp E.T., Briscoe L., Khan N., Maluk M., Lafos M., Humm E. (2018). Whole genome analyses suggests that *Burkholderia* sensu lato contains two additional novel genera (*Mycetohabitans* gen. nov., and *Trinickia* gen. nov.): Implications for the evolution of diazotrophy and nodulation in the *Burkholderiaceae*. Genes.

[95] Lopes-Santos L., Castro D.B.A., Ferreira-Tonin M., Corrêa D.B.A., Weir B.S., Park D., Ottoboni L.M.M., Neto J.R., Destéfano S.A.L. (2017). Reassessment of the taxonomic position of *Burkholderia andropogonis* and description of *Robbsia andropogonis* gen. nov., comb. nov. Antonie van Leeuwenhoek.

[96] Klemke F., Beyer G., Sawade L., Saitov A., Korte T., Maldener I., Lockau W., Nürnberg D., Volkmer T. (2014). All1371 is a polyphosphate-dependent glucokinase in *Anabaena* sp. PCC 7120. Microbiology.

[97] Francke C., Postma P., Westerhoff H., Blom J., Peletier M. (2013). Why the phosphotransferase system of *Escherichia coli* escapes diffusion limitation?. Biophys. J..

[98] Jeckelmann J.-M., Harder D., Mari S.A., Meury M., Ucurum Z., Muller D.J., Erni B., Fotiadis D. (2011). Structure and function of the glucose PTS transporter from *Escherichia coli*. J. Struct. Biol..

[99] Wilkes R.A., Mendonca C.M., Aristilde L. (2018). A Cyclic Metabolic Network in *Pseudomonas* protegens Pf-5 prioritizes the Entner-Doudoroff pathway and exhibits substrate hierarchy during carbohydrate co-utilization. Appl. Environ. Microbiol..

[100] Solhtalab M., Karbalaei-Heidari H.R., Absalan G. (2015). Tuning of hydrophilic ionic liquids concentration: A way to prevent enzyme instability. J. Mol. Catal. B Enzym..

[101] Pastor J.M., Borges N., Pagán J.P., Castaño-Cerezo S., Csonka L.N., Goodner B.W., Reynolds K.A., Gonçalves L.G., Argandoña M., Nieto J.J. (2019). Fructose metabolism in *Chromohalobacter salexigens*: Interplay between the Embden–Meyerhof–Parnas and Entner–Doudoroff pathways. Microb. Cell Fact..

[102] Fuhrer T., Fischer E., Sauer U. (2005). Experimental Identification and quantification of glucose metabolism in seven bacterial species. J. Bacteriol..

[103] Klingner A., Bartsch A., Dogs M., Wagner-Döbler I., Jahn D., Simon M., Brinkhoff T., Becker J., Wittmann C. (2015). Large-scale 13C flux profiling reveals conservation of the Entner-Doudoroff Pathway as a glycolytic strategy among marine bacteria that use glucose. Appl. Environ. Microbiol..

[104] Jyoti P., Shree M., Joshi C., Prakash T., Ray S.K., Satapathy S.S., Masakapalli S.K. (2020). The Entner-Doudoroff and nonoxidative pentose phosphate pathways bypass glycolysis and the oxidative pentose phosphate pathway in *Ralstonia solanacearum*. mSystems.

[105] Stincone A., Prigione A., Cramer T., Wamelink M.M.C., Campbell K., Cheung E., Olin-Sandoval V., Greuning N.-M., Krueger A., Alam M.T. (2015). The return of metabolism: Biochemistry and physiology of the pentose phosphate pathway. Biol. Rev..

[106] Nikel P.I., Chavarría M., Fuhrer T., Sauer U., De Lorenzo V. (2015). *Pseudomonas putida* KT2440 strain metabolizes glucose through a cycle formed by enzymes of the Entner-Doudoroff, Embden-Meyerhof-Parnas, and pentose phosphate pathways. J. Biol. Chem..

[107] Chavarría M., Nikel P.I., Pérez-Pantoja D., De Lorenzo V. (2013). The Entner-Doudoroff pathway empowers *Pseudomonas putida* KT2440 with a high tolerance to oxidative stress. Environ. Microbiol..

[108] Lemire J., AlHasawi A., Appanna V., Tharmalingam S. (2017). Metabolic defense against oxidative stress: The road less travelled so far. J. Appl. Microbiol..

[109] Obruca S., Sedlacek P., Koller M., Kucera D., Pernicová I. (2018). Involvement of polyhydroxyalkanoates in stress resistance of microbial cells: Biotechnological consequences and applications. Biotechnol. Adv..

[110] Ponce B.L., Latorre V.K., González M., Seeger M. (2011). Antioxidant compounds improved PCB-degradation by *Burkholderia xenovorans* strain LB400. Enzym. Microb. Technol..

[111] Kivisaar M. (2018). The Effect of cellular redox status on the evolvability of new catabolic pathways. mBio.

[112] Rodríguez-Castro L., Méndez V., Durán R.E., Seeger M. (2019). Long-chain flavodoxin FldX1 improves *Paraburkholderia xenovorans* LB400 tolerance to oxidative stress caused by paraquat and H_2_O_2_. PLoS ONE.

[113] Sacomboio E.N.M., Kim E.Y.S., Correa H.L.R., Bonato P., Pedrosa F.D.O., De Souza E.M., Chubatsu L.S., Müller-Santos M. (2017). The transcriptional regulator NtrC controls glucose-6-phosphate dehydrogenase expression and polyhydroxybutyrate synthesis through NADPH availability in *Herbaspirillum seropedicae*. Sci. Rep..

[114] Orellana R., Macaya C., Bravo G., Dorochesi F., Cumsille A., Valencia R., Rojas C., Seeger M. (2018). Living at the frontiers of life: Extremophiles in chile and their potential for bioremediation. Front. Microbiol..

[115] Stephens C., Christen B., Fuchs T., Sundaram V., Watanabe K., Jenal U. (2007). Genetic analysis of a novel pathway for d-xylose metabolism in *Caulobacter crescentus*. J. Bacteriol..

[116] Zhao J., Binns A.N. (2011). Characterization of the mmsAB-araD1 (gguABC) Genes of *Agrobacterium tumefaciens*. J. Bacteriol..

[117] Watanabe S., Shimada N., Tajima K., Kodaki T., Makino K. (2006). Identification and characterization of L-arabonate dehydratase, l-2-keto-3-deoxyarabonate dehydratase, and l-arabinolactonase involved in an alternative pathway of L-arabinose metabolism: Novel evolutionary insight into sugar metabolism. J. Biol. Chem..

[118] Watanabe S., Yamada M., Ohtsu I., Makino K. (2007). α-Ketoglutaric Semialdehyde Dehydrogenase isozymes involved in metabolic pathways of d-glucarate, d-galactarate, and hydroxy-l-proline. J. Biol. Chem..

[119] Ribeiro P.L.L., da Silva A.C.M.S., Filho J.A.M., Druzian J.I. (2015). Impact of different by-products from the biodiesel industry and bacterial strains on the production, composition, and properties of novel polyhydroxyalkanoates containing achiral building blocks. Ind. Crop. Prod..

[120] Sacco L.P., Castellane T.C.L., Lopes E., Lemos E.G.D.M., Alves L.M.C. (2015). Properties of polyhydroxyalkanoate granules and bioemulsifiers from *Pseudomonas* sp. and *Burkholderia* sp. isolates growing on glucose. Appl. Biochem. Biotechnol..

[121] Nomura C.T., Taguchi K., Gan Z., Kuwabara K., Tanaka T., Takase K., Doi Y. (2005). Expression of 3-ketoacyl-acyl carrier protein Reductase (fabG) genes enhances production of polyhydroxyalkanoate copolymer from glucose in recombinant *Escherichia coli* JM109. Appl. Environ. Microbiol..

[122] Scarsdale J.N., Kazanina G., He X., Reynolds K.A., Wright H.T. (2001). Crystal Structure of the *Mycobacterium tuberculosis* β-ketoacyl-acyl carrier protein synthase III. J. Biol. Chem..

[123] Röttig A., Steinbüchel A. (2013). Acyltransferases in bacteria. Microbiol. Mol. Biol. Rev..

[124] Rehm B.H.A., Mitsky T.A., Steinbuchel A. (2001). Role of fatty acid de novo biosynthesis in polyhydroxyalkanoic acid (PHA) and rhamnolipid synthesis by *Pseudomonads*: Establishment of the transacylase (PhaG)-mediated pathway for PHA biosynthesis in *Escherichia coli*. Appl. Environ. Microbiol..

[125] Zheng Z., Chen J.-C., Tian H.-L., Bei F.-F., Chen G.-Q. (2005). Specific identification of (R)-3-hydroxyacyl-ACP: CoA transacylase gene from *Pseudomonas* and *Burkholderia* strains by polymerase chain reaction. Sheng Wu Gong Cheng Xue Bao.

[126] Davis R., Chandrashekar A., Shamala T.R. (2007). Role of (R)-specific enoyl coenzyme A hydratases of *Pseudomonas* sp in the production of polyhydroxyalkanoates. Antonie van Leeuwenhoek.

[127] Tsuge T., Taguchi S., Seiichi T., Doi Y. (2003). Molecular characterization and properties of (R)-specific enoyl-CoA hydratases from *Pseudomonas aeruginosa*: Metabolic tools for synthesis of polyhydroxyalkanoate via fatty acid beta-oxidation. Int. J. Biol. Macromol..

[128] Rehm B.H.A. (2006). Genetics and biochemistry of polyhydroxyalkanoate granule self-assembly: The key role of polyester synthases. Biotechnol. Lett..

[129] Snell K., Feng F., Zhong L., Martin D., Madison L. (2002). YfcX enables medium-chain-length poly(3-hydroxyalkanoate) formation from fatty acids in recombinant *Escherichia coli fadB* strains. J. Bacteriol..

[130] Fujita Y., Matsuoka H., Hirooka K. (2007). Regulation of fatty acid metabolism in bacteria. Mol. Microbiol..

[131] Riedel S.L., Lu J., Stahl U., Brigham C.J. (2014). Lipid and fatty acid metabolism in *Ralstonia eutrop*h*a*: Relevance for the biotechnological production of value-added products. Appl. Microbiol. Biotechnol..

[132] Slater S., Houmiel K.L., Tran M., Mitsky T.A., Taylor N.B., Padgette S.R., Gruys K.J. (1998). Multiple β-ketothiolases mediate poly(β-hydroxyalkanoate) copolymer synthesis in *Ralstonia eutropha*. J. Bacteriol..

[133] Rand J.M., Pisithkul T., Clark R.L., Thiede J.M., Mehrer C.R., Agnew D.E., Campbell C.E., Markley A.L., Price M.N., Ray J. (2017). A metabolic pathway for catabolizing levulinic acid in bacteria. Nat. Microbiol..

[134] Chee J.-Y., Lau N.-S., Samian M.-R., Tsuge T., Sudesh K. (2011). Expression of *Aeromonas caviae* polyhydroxyalkanoate synthase gene in *Burkholderia* sp. USM (JCM15050) enables the biosynthesis of SCL-MCL PHA from palm oil products. J. Appl. Microbiol..

[135] Mezzolla V., D’Urso O.F., Poltronieri P. (2018). Role of PhaC type i and type ii enzymes during PHA biosynthesis. Polymers.

[136] Wittenborn E.C., Jost M., Wei Y., Stubbe J., Drennan C.L. (2016). Structure of the catalytic domain of the class i polyhydroxybutyrate synthase from *Cupriavidus necator*. J. Biol. Chem..

[137] Rehm B.H.A. (2003). Polyester synthases: Natural catalysts for plastics. Biochem. J..

[138] Gradíssimo D.G., Xavier L.P., Santos A.V. (2020). Cyanobacterial Polyhydroxyalkanoates: A sustainable alternative in circular economy. Molecules.

[139] Jendrossek D., Pfeiffer D. (2014). New insights in the formation of polyhydroxyalkanoate granules (carbonosomes) and novel functions of poly(3-hydroxybutyrate). Environ. Microbiol..

[140] Tsuge T., Hyakutake M., Mizuno K. (2015). Class IV polyhydroxyalkanoate (PHA) synthases and PHA-producing *Bacillus*. Appl. Microbiol. Biotechnol..

[141] Rodrigues M.F.D.A., Vicente E.J., Steinbüchel A. (2000). Studies on polyhydroxyalkanoate (PHA) accumulation in a PHA synthase I-negative mutant of *Burkholderia cepacia* generated by homogenotization. FEMS Microbiol. Lett..

[142] A Chen I.-M., Chu K., Palaniappan K., Pillay M., Ratner A., Huang J., Huntemann M., Varghese N., White J.R., Seshadri R. (2019). IMG/M v.5.0: An integrated data management and comparative analysis system for microbial genomes and microbiomes. Nucleic Acids Res..

[143] Tan I.K.P., Foong C.P., Tan H.T., Lim H., Zain N.-A.A., Tan Y.C., Hoh C.C., Sudesh K. (2020). Polyhydroxyalkanoate (PHA) synthase genes and PHA-associated gene clusters in *Pseudomonas* spp. and *Janthinobacterium* spp. isolated from Antarctica. J. Biotechnol..

[144] Kim J., Kim Y.-J., Choi S.Y., Lee S.Y., Kim K.-J. (2017). Crystal structure of *Ralstonia eutropha* polyhydroxyalkanoate synthase C-terminal domain and reaction mechanisms. Biotechnol. J..

[145] Hiroe A., Tsuge K., Nomura C.T., Itaya M., Tsuge T. (2012). Rearrangement of gene order in the *phaCAB* operon leads to effective production of ultrahigh-molecular-weight poly[(R)-3-hydroxybutyrate] in genetically engineered *Escherichia coli*. Appl. Environ. Microbiol..

[146] Mezzina M.P., Pettinari M.J. (2016). Phasins, Multifaceted polyhydroxyalkanoate granule-associated proteins. Appl. Environ. Microbiol..

[147] Park S.J., Lee S.Y. (2003). Identification and characterization of a new enoyl coenzyme a hydratase involved in biosynthesis of medium-chain-length polyhydroxyalkanoates in recombinant *Escherichia coli*. J. Bacteriol..

[148] Wolfe A.J. (2005). The Acetate Switch. Microbiol. Mol. Biol. Rev..

[149] Chen J., Li W., Zhang Z.-Z., Tan T.-W., Li Z.-J. (2018). Metabolic engineering of *Escherichia coli* for the synthesis of polyhydroxyalkanoates using acetate as a main carbon source. Microb. Cell Fact..

[150] Summers M.L., Denton M.C., McDermott T.R. (1999). Genes coding for phosphotransacetylase and acetate kinase in *Sinorhizobium meliloti* are in an operon that is inducible by phosphate stress and controlled by phob. J. Bacteriol..

[151] Morris J., Fane A., Rush C., Govan B., Mayo M., Currie B.J., Ketheesan N. (2015). Neurotropic threat characterization of *Burkholderia pseudomallei* strains. Emerg. Infect. Dis..

[152] Price E.P., MacHunter B., Spratt B.G., Wagner D.M., Currie B.J., Sarovich D.S. (2016). Improved multilocus sequence typing of *Burkholderia pseudomallei* and closely related species. J. Med. Microbiol..

[153] Haq I.U., Zwahlen R.D., Yang P., Van Elsas J.D. (2018). The response of *Paraburkholderia* terrae strains to two soil fungi and the potential role of oxalate. Front. Microbiol..

[154] Goris J., Dejonghe W., Falsen E., De Clerck E., Geeraerts B., Willems A., Top E.M., Vandamme P., De Vos P. (2002). Diversity of transconjugants that acquired plasmid pjp4 or pEMT1 after Inoculation of a donor strain in the a- and b-horizon of an agricultural soil and description of *Burkholderia hospita* sp. nov. and *Burkholderia terricola* sp. nov. Syst. Appl. Microbiol..

[155] Liu X.-Y., Li C.-X., Luo X.-J., Lai Q.-L., Xu J.-H. (2014). *Burkholderia jiangsuensis* sp. nov., a methyl parathion degrading bacterium, isolated from methyl parathion contaminated soil. Int. J. Syst. Evol. Microbiol..

[156] Baek I., Seo B., Lee I., Yi H., Chun J. (2015). *Burkholderia monticola* sp. nov., isolated from mountain soil. Int. J. Syst. Evol. Microbiol..

[157] Pötter M., Madkour M.H., Mayer F., Steinbüchel A. (2002). Regulation of phasin expression and polyhydroxyalkanoate (PHA) granule formation in *Ralstonia eutropha* H16. Microbiology.

[158] Bailey T.L., Johnson J., Grant C.E., Noble W.S. (2015). The MEME Suite. Nucleic Acids Res..

[159] Nguyen L.-T., Schmidt H.A., Von Haeseler A., Minh B.Q. (2015). IQ-TREE: A fast and effective stochastic algorithm for estimating maximum-likelihood phylogenies. Mol. Biol. Evol..

[160] Kalyaanamoorthy S., Minh B.Q., Wong T.K.F., von Haeseler A., Jermiin L.S. (2017). ModelFinder: Fast model selection for ac-curate phylogenetic estimates. Nat. Methods.

[161] Hoang D.T., Chernomor O., von Haeseler A., Minh B.Q., Vinh L.S. (2018). UFBoot2: Improving the ultrafast bootstrap approximation. Mol. Biol. Evol..

[162] Katoh K., Rozewicki J., Yamada K.D. (2019). MAFFT online service: Multiple sequence alignment, interactive sequence choice and visualization. Briefings Bioinform..

[163] Letunic I., Bork P. (2019). Interactive Tree of Life (iTOL) v4: Recent updates and new developments. Nucleic Acids Res..

