# Supplementary material for: Genome-Wide Metabolic Reconstruction of the Synthesis of Polyhydroxyalkanoates from Sugars and Fatty Acids by Burkholderia Sensu Lato Species
Source: Microorganisms. 2021 Jun 12;9(6):1290. doi: 10.3390/microorganisms9061290 (PMC8231600; doi:10.3390/microorganisms9061290)
Supplement: Supplementary file 1 [file microorganisms-09-01290-s001.zip › microorganisms-1212438-supplementary.pdf]

Supplementary Material

Supplementary Table

Table S1. Assimilation of substrates commonly used for PHA production in 37 *Burkholderia* sensu lato type and representative strains.

Strain	Substrate assimilation								Reference
	Scr	Man	Glu	Fru	Xyl	Ara	Gln	Gly	
Burkholderia cepacia ATCC 25416^T	+	+	+	+	+	+	+	+	[1]
Burkholderia contaminans MS14	nr	nr	nr	nr	nr	nr	nr	nr	-
Burkholderia cenocepacia J2315	+	+	+	nr	+	nr	nr	nr	[2]
Burkholderia stabilis ATCC BAA-67^T	-	+	+	nr	nr	+	nr	nr	[3]
Burkholderia pyrrocinia DSM 10685^T	+	nr	nr	+	nr	+	nr	nr	[4]
Burkholderia vietnamiensis LMG 10929^T	+	+	+	+	+	+	nr	+	[5]
Burkholderia ambifaria AMMD^T	nr	+	+	nr	+	+	+	nr	[6]
Burkholderia stagnalis LMG 28156^T	+	+	nr	nr	+	+	nr	nr	[7]
Burkholderia multivorans ATCC 17616	-	+	+	+	+	+	-	+	[2,8]
Burkholderia thailandensis E264^T	nr	nr	nr	nr	nr	+	nr	+	[9,10]
Burkholderia mallei ATCC 23344^T	+	+	+	-	-	-	+	+	[5,9,11]
Burkholderia glumae LMG 2196^T	+	+	+	+	+	+	+	-	[1]
Burkholderia plantarii ATCC 43733^T	-	+	+	+	+	+	nr	+	[1]
Paraburkholderia graminis PHS1	+	nr	nr	+	nr	+	nr	nr	[4]
Paraburkholderia caledonica PHRS4	-	nr	+	+	+	+	nr	nr	[6]
Paraburkholderia aromaticivorans BN5^T	+	+	+	+	nr	+	+	+	[12]
Paraburkholderia xenovorans LB400^T	-	+	+	+	+	-	+	nr	[13]
Paraburkholderia phytofirmans PsJN^T	+	nr	+	+	+	nr	nr	nr	[14]
Paraburkholderia fungorum ATCC BAA-463^T	-	nr	+	+	+	+	nr	nr	[6]

Paraburkholderia caffeinilytica CF1^{T}	-	-	-	+	+	+	-	+	[15]
Paraburkholderia sprentiae WSM5005^{T}	nr	+	+	nr	nr	+	+	nr	[13]
Paraburkholderia megapolitana LMG 23650^{T}	+	+	+	nr	nr	+	+	nr	[8,13]
Paraburkholderia terrae DSM 17804^{T}	-	+	+	nr	nr	+	+	nr	[16]
Paraburkholderia hospita DSM 17164^{T}	-	+	+	nr	-	+	+	nr	[13]
Paraburkholderia phymatum STM 815^{T}	nr	+	+	nr	nr	+	+	nr	[13]
Paraburkholderia sacchari LMG 19450^{T}	+	+	+	+	+	+	+	+	[4]
Paraburkholderia tropica LMG 22274^{T}	-	+	+	+	+	+	+	+	[13]
Caballeronia sordidicola LMG 22029	-	+	+	-	nr	+	+	-	[17]
Caballeronia udeis LMG 27134^{T}	-	+	+	+	nr	+	+	-	[17]
Caballeronia glathei LMG:14190^{T}	-	+	+	+	nr	+	+	-	[17]
Caballeronia insecticola RPE64^{T}	nr	+	+	nr	nr	+	+	nr	[18]
Caballeronia cordobensis LMG 27620^{T}	-	+	nr	nr	nr	nr	+	nr	[19]
Caballeronia grimmiae LMG 27580^{T}	nr	+	+	nr	nr	+	+	nr	[13]
Trinickia caryophylli LMG 2155^{T}	+	-	+	+	+	+	nr	+	[1]
Trinickia symbiotica JPY-345^{T}	nr	+	+	+	nr	+	nr	nr	[20]
Mycetohabitans rhizoxinica HKI 454^{T}	nr	nr	-	nr	nr	nr	nr	+	[20]
Robbsia andropogonis LMG 2129^{T}	-	+	nr	+	nr	+	nr	nr	[21]

Substrates claimed to be assimilated (+) or not (-) and those not found in literature (not reported: nr). Scr: sucrose; Man: mannitol; Glu: *D*-glucose; Fru: *D*-fructose; Xyl: *D*-Xylose; Ara: *L*-Arabinose; Gln: Gluconate; Gly: glycerol.

Supplementary Figures

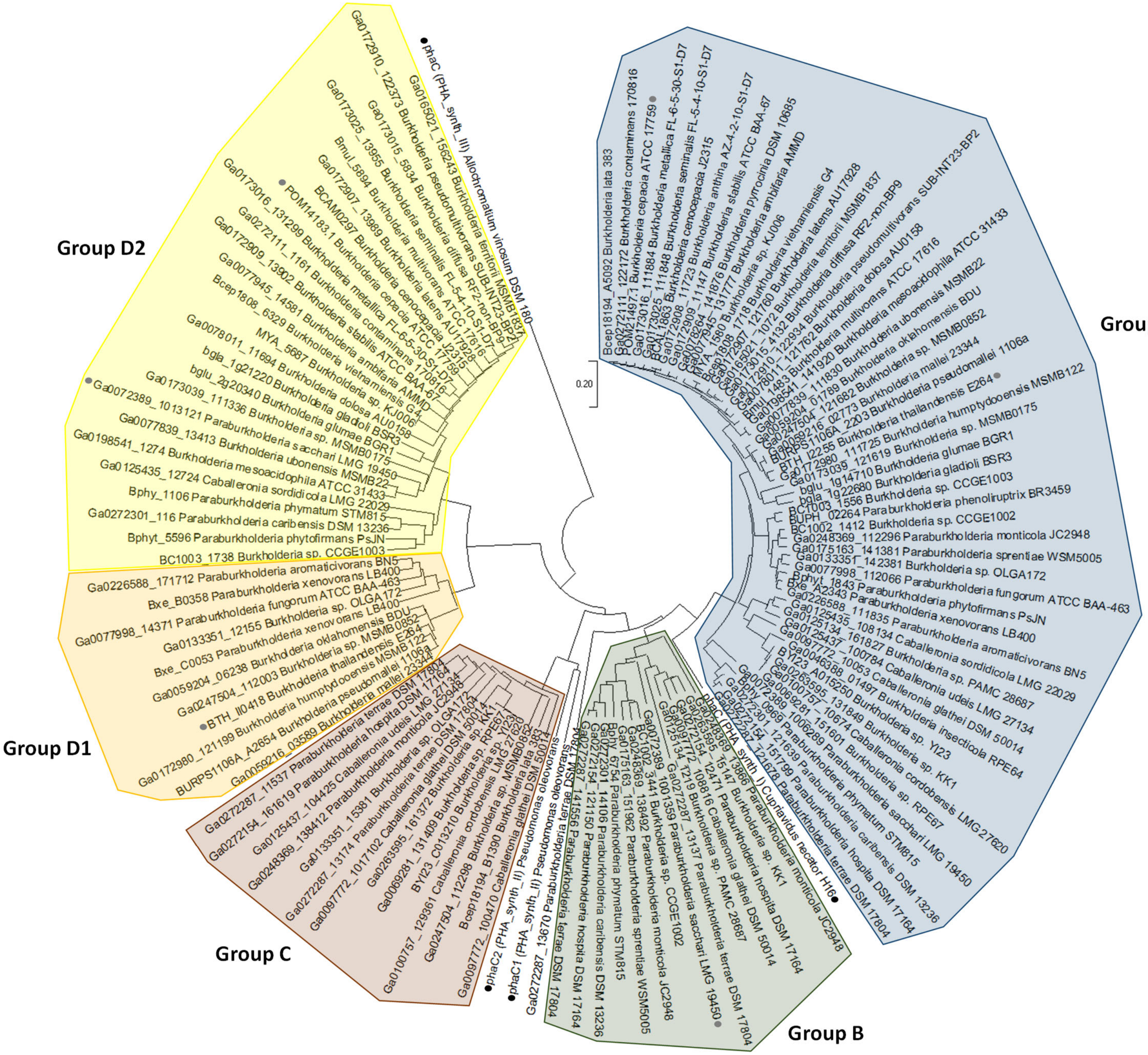

Supplementary Figure 1. Phylogenetic tree for PhaC of representative *Burkholderia* sensu lato species. The phylogenetic tree was obtained by the neighbor joining method using MEGA-X software based on sequence alignments calculated by CLUSTALW. PhaC sequences were retrieved from IMG/M database considering similarity by the chance expectation values and scores of BLAST probing. Confidence in phylogenetic inference was assessed using non-parametric bootstrap resampling.

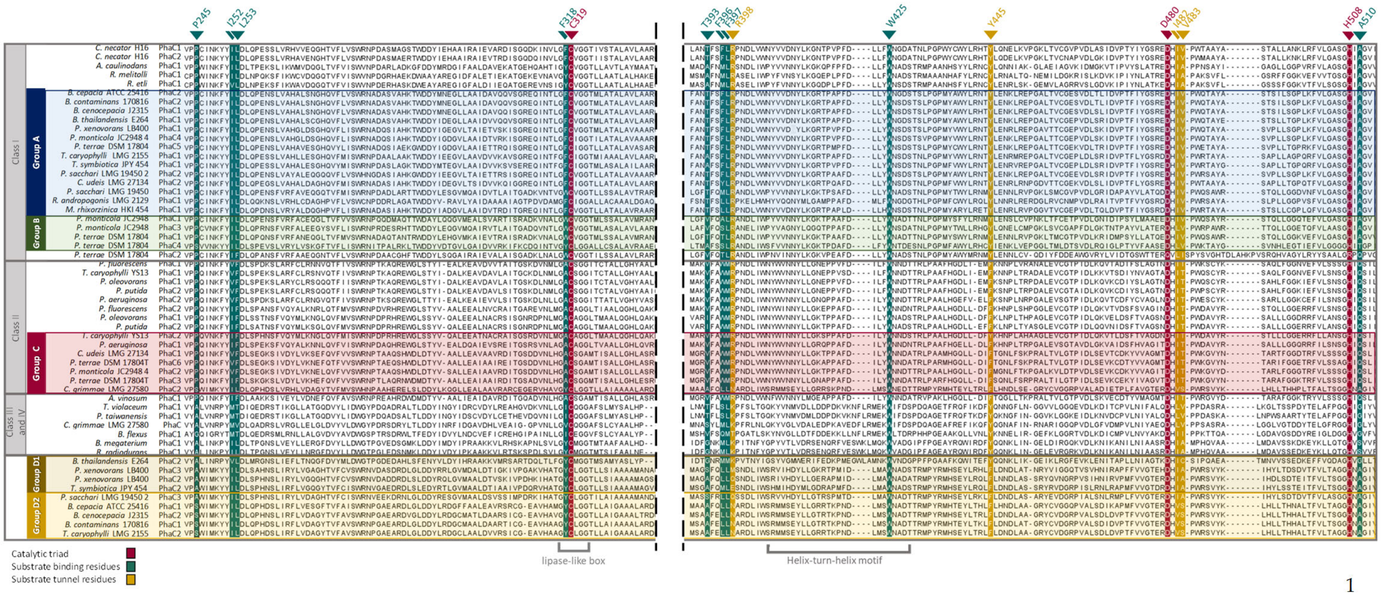

Supplementary Figure 2. Multiple sequence alignment of PHA synthases of four phylogenetic groups in *Burkholderia* sensu lato genomes. The conserved amino acidic residues and the lipase box-like identified in PHA synthases sequences are highlighted. PHA synthases of class I, II, III and IV are included as reference. The alignment was made using MAFFT.

Motif Logo

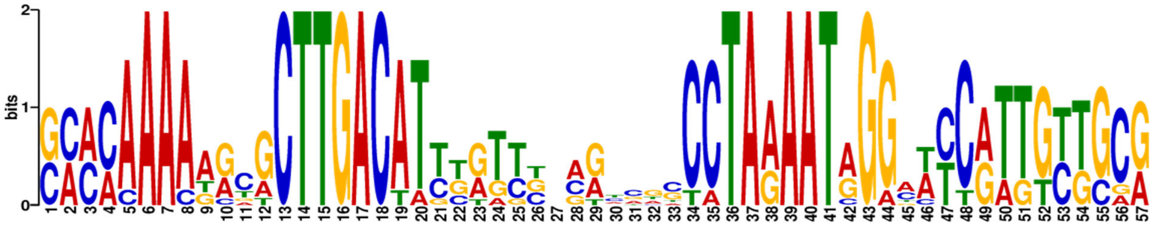

Motif Consensus

SMMMAAAAARCGCTTGACATYBRTYBNVRHVDSCCTARAATRGGMWYCRTTGYKGCR

>CP039287.1:c1498517-1498218 ***Cupriavidus necator* H16**

GGGCGCGGGCAATCCGCGATGGTAGCACCCCTGCATGGCCCTCGCCGGAGCGCCCCGGAGTGGC

GTCACAGCCGCTCCCGTGTATCGCCAGCAACGTTGTTTGTGCATT**GCACAAAATCCACTTGACA**

TTGGATCTGGCGCCCCTAAAATAGGAATTGTTGCGGCGCACCAAATAAGAAATGCGCCTTGACC

CACCCAACGCCTGGGCTGGCCGAATCGGGCACAACACCGTCACGGCCCTGACATCTAGGCGGCT

TAATTTGCTAGACCTTGAAGTTCACCACTGGAGACCAGCA**ATG**

>CP000270.1:3203820-3204119 ***Paraburkholderia xenovorans* LB400**

GCCCATCTAGCGGTAATAAACCGACAAATATGTCGCACCAGCACGGTGCAACGCGGAAATTGAT
CTGCGTCACATGAACGTCATGCCCAACGCATAACGTCTTGTTTTATCAGTAGTTTCTTAATATG
GTGCGACGCACAAAAAAGGACTTGACATTCGGTGCAGCCATCCTAAAATGGGAACCATTGCTGC
GATGCACAAAACATCGCGGTCCGGCGGGGCGCATCACTATCAATGTGTCTCACCACTCACCCAA
TGCGGTCCGCACAGACAGACCGCGATCCAGGAGCGTAAACC**ATG**

>JTDB02000008.1:c192093-191794 **Paraburkholderia sacchari LMG 19450**
TGCGCTTGACGTTGTCGGACAAAGCCTCGCCGCACGCAGGCTTCAGACGCTATGCCGCAGATCG
TCAAATCTGACAAATGACCTCATACACCTCATGCTTGACGCATAAGTAATTGTTTAATAAAAGC
TTAATGTAATTGTGCGTCGCACCAAAAGTTCTTGACATTCATCTCCGGCGACCTAAAATCGAGA
TCAATGCTGCGACGCACCAAAGCGCCGGGCAGATTCCCCCAAGAGTCTTGCCACTTACTTCAGG
CGGCCCACGAACGAGGCCGCCAAACATCCAGGAGCGTAAAC**ATG**

>CP000868.1:c1255064-1254765 **Burkholderia multivorans ATCC 17616**
AACGTTCCAGGCGCGTGCGCGGTTTACCTGCGCGCGATCATGGTGCGCCGCGCGCCGGGGCACG
CGCCGTGCACCAGCGTGGACACCCACTGAAACCGATTCCGGAACGCGGCGGTAAGCCGTTGTTT
TGTCGATAATTTGTCGTGATTGTGCAATGCACAAAAAATGCTTGACAAGGTTTTTCTCTGCCCT
AAAATAGGCTCCATTGCTGCGTTGCACAAAGCACGTCGCCGCAAGATTCCCCAGCGCAGTGCCC
TTTACCCCGCGATCGACGCGATCGCTTTCCAGGAGCTTGAC**ATG**

>CP009435.1:3550343-3550642 **Burkholderia glumae LMG 2196**
AAACCCGCGATGGGCGCGCTTTGCCAAGGGCGTCGCGAGTCAGGCGCATCGAGCCGTCTCATGC
AATGCCGGCGGACCGGAACCGATTTCATCCGGAACGATACAAGCCATTGTTTTTCCGGAAAAAT
AGTGTCATTGTGCAGCGCACAAAAGAAGCTTGACATCTGTTTCGAACGCCCTAGAATGGGAACC
GTTGTTGCAATGCACCACGGCATTGCGCAGATTCCGAACACCGTCGTGCCCCTTAACTCAAGCG
ATCGCCGCGAAGCCGGCGATCGTCAATCAGGAGCCAAAATC**ATG**

>JFHE01000079.1:6667-6966 **Caballeronia grimmiae LMG 27580**
CAACATGCGCCAACGCAGCCGCGCGCAGCATGGGACTCGCCGCTTTTCGCTAGTCCACGTCGGT
GCATGCGCCGCCCGGTTCACGCCCTTTCCGACCGAATGCCGTAGTTAAACTCTTGTTTAACAAG
GGTTTCCCCTATTTGTGCATTGCACAAAAAGCGCTTGACATCGATCGGAATATCTCTAAAATGG
GCTTCATGTTGCAACGCACCAATCCGGAGCTGCACCATCTGGCAGCAAGTTGCTGCATCTACCA
AACTACGACCCGCGATGATGGGTCGGCATAAGGAGCCTGAG**ATG**

>FCOK02000074.1:c21237-20938 **Caballeronia udeis LMG 27134**
CCGGACTTGGGATCCTAGCATCGACACCGCCTGCGTTCTCCGCCCCAAGCTGGTGCACCGTATC
TCGAAATGTAGTTCCACTACAAAGAAGTGTCCTCGTAAGCCATTGTTTAATAAGCATTCGTACG
ATGTTGCGTCGCACAAAAAGACGCTTGACATCTATCGGAATATGCCTAGAATAGGCTCCATGTT
GCGACGCACCAATAGCTGATCGCCTCATACGGTTTCCGGATTTCCGGTCTCCGCCAAAACCCAC
GACCCGCGCTGATGATGGGTCGAATCTTTCAGGAGCGTGAC**ATG**

>VJZF01000001.1:c156642-156343 **Trinickia caryophylli DSM 50341**
AGGAAGGCCGCCATCGAAGGGTCCGTCGCCCTTTCAGCGCGACGCAGCAAAATCCATTCCAAGC
GCCCTTCGCCGCTGCCCTACCATTCCTCGTAGCACCTTGTTTTTATGCAGAATCCTTTGAATTA
GACAGAATTGTGCATCGCACCAAACAGCGCTTGACTTTTGTGCACGACGGCATAGAATAGGTCT

CGATGTTGCGACGCACAAAAGGCGTCGGGCATACGACGCGATTCGAAGCTTTGCCATTAACCTA
TGCGATCTGCGCCAGACAGGTCGATTTTCAGGAGCGAAAAC**ATG**
>PTIR01000018.1:c93163-92864 ***Trinickia symbiotica* JPY-345**
GGCGGGAGCGTGGGCGCGATCGTATCACGAGACTGGTGCACGAGGGCGAAGCACTGCCGCATTT
TTTCCCGGAAAGCGAGCGTTTGCTCGGCCAAAATTACCGTCATGCCGTTGTTTTTACGTGGAAA
CCCTAACATTGTGCAAT**GCACAAAAATGCGCTTGACATTTGGTCAAGTGAGCCTAAAATAGGAT**
CCGTTGTTGCGACGCACAAAACGCGTCGGCTAGACGACGCGATCAAAGTTTTGCCATTCCACCC
CCCGGTCTGCGCCAGACAGGCCGACTATCAGGAGCGAAAAC**ATG**

Supplementary Figure 3. Conserved motif in the putative promoter sequence of the *phaP* gene in 8 selected *Burkholderia* sensu lato strains and the model PHA-producer *C. necator* H16. The 300 bp *phaP* upstream regions, containing putative regulatory sequences, were recovered from 8 genomes of *Burkholderia* s.l. strains, including two species per main genera (*Burkholderia*, *Paraburkholderia*, *Caballeronia* and *Trinickia*), and from *C. necator* H16 genome, in which the repression of *phaP* expression by the PhaR regulator has been reported [22], and altogether used for a consensus motif search. The web-based MEME suite of motif-based sequence analysis tools version 5.3.3 [23] was used for motif discovery utilizing standard settings. The logo and consensus sequence of a 57 bp-motif identified in the nine putative regulatory regions included is shown in the top. The 300 bp *phaP* upstream region of each strain is also shown including every motif (in color) and the ATG initiation codon of each gene (in bold inside the box). The PhaR binding sites determined by DNase I footprinting experiments in *C. necator* H16 [22] are also shown in gray boxes. The accession numbers and coordinates of each sequence are included in the header of the fasta format

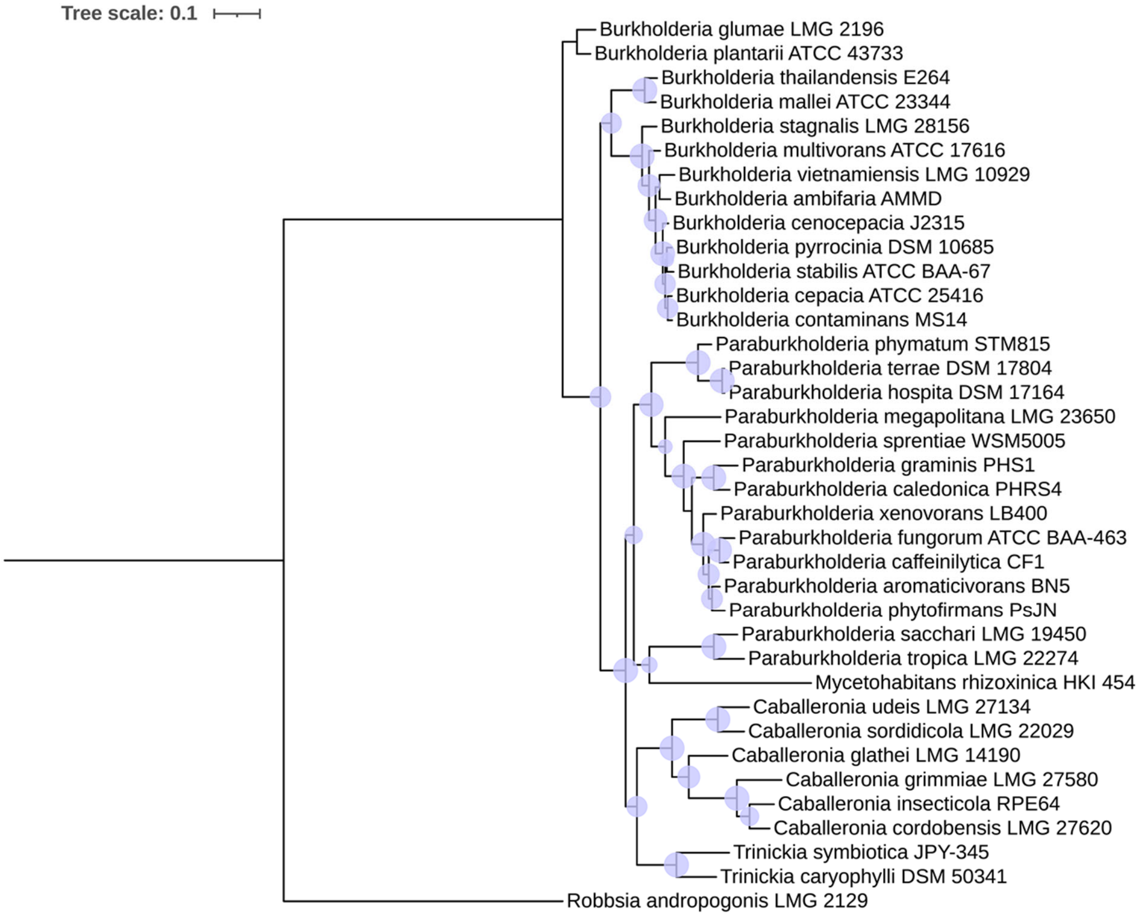

Supplementary Figure 4. Evolutionary relationships among concatenated PhaA, PhaB and PhaC protein homologues encoded by the conserved canonical *phaCAB* gene cluster of the 37 *Burkholderia* sensu lato genomes. Maximum likelihood (ML) topology provided by IQ-TREE [24] employing ModelFinder as model-selection method [25] and Ultrafast Bootstrap Approximation as bootstrap approach [26] with the -m TEST, -bb 1000 and -alrt 1000 options. Sequence alignments for phylogenetic reconstruction were calculated with MAFFT online service using Auto (FFT-NS-1, FFT-NS-2, FFT-NS-i or L-INS-i; depends on data size) strategy [27]. ML topology is shown with SH-like approximate likelihood ratio support values (n = 1000) given at each node (values >50% are shown). Visualization and edition of phylogenetic trees were performed by the Interactive Tree Of Life (iTOL) online tool [28]

References

1. Urakami, T.; Ito-Yoshida, C.; Araki, H.; Kijima, T.; Suzuki, K.-I.; Komagata, K. Transfer of Pseudomonas plantarii and *Pseudomonas glumae* to Burkholderia as *Burkholderia* spp. and description of *Burkholderia vandii* sp. nov. *Int. J. Syst. Bacteriol.* **1994**, *44*, 235–245, doi:10.1099/00207713-44-2-235.
2. Vandamme, P.; Holmes, B.; Vancanneyt, M.; Coenye, T.; Hoste, B.; Coopman, R.; Revets, H.; Lauwers, S.; Gillis, M.; Kersters, K.; et al. Occurrence of multiple genomovars of *Burkholderia cepacia* in cystic fibrosis patients and proposal of *Burkholderia multivorans* sp. nov. *Int. J. Syst. Bacteriol.* **1997**, *47*, 1188–1200, doi:10.1099/00207713-47-4-1188.
3. Seth-Smith, H.M.; Casanova, C.; Sommerstein, R.; Meinel, D.M.; Abdelbary, M.M.; Blanc, D.S.; Droz, S.; Führer, U.; Lienhard, R.; Lang, C.; et al. Phenotypic and genomic analyses of *Burkholderia stabilis* clinical contamination, Switzerland. *Emerg. Infect. Dis.* **2019**, *25*, 1084–1092, doi:10.3201/eid2506.172119.

4. O Brämer, C.; Vandamme, P.; Da Silva, L.F.; Gomez, J.G.C.; Steinbüchel, A. Polyhydroxyalkanoate-accumulating bacterium isolated from soil of a sugar-cane plantation in Brazil. *Int. J. Syst. Evol. Microbiol.* **2001**, *51*, 1709–1713, doi:10.1099/00207713-51-5-1709.
5. Gillis, M.; Van Van, T.; Bardin, R.; Goor, M.; Hebbar, P.; Willems, A.; Segers, P.; Kersters, K.; Heulin, T.; Fernandez-Fernandez, M.P. Polyphasic taxonomy in the genus *Burkholderia* Leading to an emended description of the genus and proposition of *Burkholderia vietnamiensis* sp. nov. for n2-fixing isolates from rice in Vietnam. *Int. J. Syst. Bacteriol.* **1995**, *45*, 274–289, doi:10.1099/00207713-45-2-274.
6. Coenye, T.; Laevens, S.; Willems, A.; Olén, M.; Hannat, W.; Govan, J.; Gillis, M.; Falsen, E.; Vandamme, P. *Burkholderia fungorum* sp. nov. and *Burkholderia caledonica* sp. nov., two new species isolated from the environment, animals and human clinical samples. *Int. J. Syst. Evol. Microbiol.* **2001**, *51*, 1099–1107, doi: 10.1099/00207713-45-2-274. DOI: 10.1099/00207713-51-3-1099.
7. De Smet, B.; Mayo, M.; Peeters, C.; Zlosnik, J.; Spilker, T.; Hird, T.J.; Lipuma, J.J.; Kidd, T.; Kaestli, M.; Ginther, J.L.; et al. *Burkholderia stagnalis* sp. nov. and *Burkholderia territorii* sp. nov., two novel *Burkholderia cepacia* complex species from environmental and human sources. *Int. J. Syst. Evol. Microbiol.* **2015**, *65*, 2265–2271, doi:10.1099/ijs.0.000251.
8. Vandamme, P.; Opelt, K.; Knöchel, N.; Berg, C.; Schönmann, S.; De Brandt, E.; Eberl, L.; Falsen, E.; Berg, G. *Burkholderia bryophila* sp. nov. and *Burkholderia megapolitana* sp. nov., moss-associated species with antifungal and plant-growth-promoting properties. *Int. J. Syst. Evol. Microbiol.* **2007**, *57*, 2228–2235, doi:10.1099/ijs.0.65142-0.
9. Brett, P.J.; DeShazer, D.; Woods, D.E. Note: *Burkholderia thailandensis* sp. nov., a *Burkholderia pseudomallei*-like species. *Int. J. Syst. Bacteriol.* **1998**, *48*, 317–320, doi:10.1099/00207713-48-1-317.
10. Victor, I.U.; Kwiencien, M.; Tripathi, L.; Cobice, D.; McClean, S.; Marchant, R.; Banat, I.M. Quorum sensing as a potential target for increased production of rhamnolipid biosurfactant in *Burkholderia thailandensis* E264. *Appl. Microbiol. Biotechnol.* **2019**, *103*, 6505–6517, doi:10.1007/s00253-019-09942-5.
11. Yabuuchi, E.; Kosako, Y.; Oyaizu, H.; Yano, I.; Hotta, H.; Hashimoto, Y.; Ezaki, T.; Arakawa, M. Proposal of *Burkholderia* gen. nov. and transfer of seven species of the genus *Pseudomonas* homology group ii to the new genus, with the type species *Burkholderia cepacia* (Palleroni and Holmes 1981) comb. nov. *Microbiol. Immunol.* **1992**, *36*, 1251–1275, doi:10.1111/j.1348-0421.1992.tb02129.x.
12. Lee, Y.; Jeon, C.O. *Paraburkholderia aromaticivoran*s sp. nov., an aromatic hydrocarbon-degrading bacterium, isolated from gasoline-contaminated soil. *Int. J. Syst. Evol. Microbiol.* **2018**, *68*, 1251–1257, doi:10.1099/ijsem.0.002661.
13. Barberan, A.; International journal of systematic and evolutionary microbiology (IJSEM) phenotypic database. Figshare. Dataset. **2016**. doi: 10.6084/m9.figshare.4272392. (accessed 20 January 2021).
14. Sessitsch, A.; Coenye, T.; Sturz, A.V.; Vandamme, P.; Barka, E.A.; Salles, J.F.; Van Elsas, J.D.; Faure, D.; Reiter, B.; Glick, B.R.; et al. *Burkholderia phytofirmans* sp. nov., a novel plant-associated bacterium with plant-beneficial properties. *Int. J. Syst. Evol. Microbiol.* **2005**, *55*, 1187–1192, doi:10.1099/ijs.0.63149-0.
15. Gao, Z.; Yuan, Y.; Xu, L.; Liu, R.; Chen, M.; Zhang, C. *Paraburkholderia caffeinilytica* sp. nov., isolated from the soil of a tea plantation. *Int. J. Syst. Evol. Microbiol.* **2016**, *66*, 4185–4190, doi:10.1099/ijsem.0.001333.
16. Yang, H.-C.; Im, W.-T.; Kim, K.K.; An, D.-S.; Lee, S.-T. *Burkholderia terrae* sp. nov., isolated from a forest soil. *Int. J. Syst. Evol. Microbiol.* **2006**, *56*, 453–457, doi:10.1099/ijs.0.63968-0.
17. Vandamme, P.; De Brandt, E.; Houf, K.; Salles, J.F.; Van Elsas, J.D.; Spilker, T.; LiPuma, J.J. *Burkholderia humi* sp. nov., *Burkholderia choica* sp. nov., *Burkholderia telluris* sp. nov., *Burkholderia terrestris* sp. nov. and *Burkholderia udeis* sp. nov.: *Burkholderia glathei*-like bacteria from soil and rhizosphere soil. *Int. J. Syst. Evol. Microbiol.* **2013**, *63*, 4707–4718, doi:10.1099/ijs.0.048900-0.
18. Takeshita, K.; Tamaki, H.; Ohbayashi, T.; Meng, X.-Y.; Sone, T.; Mitani, Y.; Peeters, C.; Kikuchi, Y.; Vandamme, *P. Burkholderia insecticola* sp. nov., a gut symbiotic bacterium of the bean bug *Riptortus pedestris*. *Int. J. Syst. Evol. Microbiol.* **2018**, *68*, 2370–2374, doi:10.1099/ijsem.0.002848.
19. Draghi, W.O.; Peeters, C.; Cnockaert, M.; Snauwaert, C.; Wall, L.G.; Zorreguieta, A.; Vandamme, P. *Burkholderia cordobensis* sp. nov., from agricultural soils. *Int. J. Syst. Evol. Microbiol.* **2014**, *64*, 2003–2008, doi:10.1099/ijs.0.059667-0.
20. Santos, P.E.-D.L.; Palmer, M.; Chávez-Ramírez, B.; Beukes, C.; Steenkamp, E.T.; Briscoe, L.; Khan, N.; Maluk, M.; Lafos, M.; Humm, E.; et al. whole genome analyses suggests that *Burkholderia* sensu lato contains two additional novel genera (*Mycetohabitans* gen. nov., and *Trinickia* gen. nov.): implications for the evolution of diazotrophy and nodulation in the *Burkholderiaceae*. *Genes* **2018**, *9*, 389, doi:10.3390/genes9080389.
21. Lopes-Santos, L.; Castro, D.B.A.; Ferreira-Tonin, M.; Corrêa, D.B.A.; Weir, B.S.; Park, D.; Ottoboni, L.M.M.; Neto, J.R.; Destéfano, S.A.L. Reassessment of the taxonomic position of *Burkholderia andropogonis* and description of *Robbsia andropogonis* gen. nov., comb. nov. *Antonie van Leeuwenhoek* **2017**, *110*, 727–736, doi:10.1007/s10482-017-0842-6.
22. Pötter, M.; Madkour, M.H.; Mayer, F.; Steinbüchel, A. Regulation of phasin expression and polyhydroxyalkanoate (PHA) granule formation in *Ralstonia eutropha* H16. *Microbiology* **2002**, *148*, 2413–2426, doi:10.1099/00221287-148-8-2413.
23. Bailey, T.L.; Johnson, J.; Grant, C.E.; Noble, W.S. The MEME Suite. *Nucleic Acids Res.* **2015**, *43*, W39–W49, doi:10.1093/nar/gkv416.
24. Nguyen, L.-T.; Schmidt, H.A.; Von Haeseler, A.; Minh, B.Q. IQ-TREE: A fast and effective stochastic algorithm for estimating maximum-likelihood phylogenies. *Mol. Biol. Evol.* **2015**, *32*, 268–274, doi:10.1093/molbev/msu300.
25. Kalyaanamoorthy, S.; Minh, B.Q.; Wong, T.K.F.; von Haeseler, A.; Jermiin, L.S. ModelFinder: fast model selection for ac-curate phylogenetic estimates. *Nat. Methods* **2017**, *14*, 587–589.
26. Hoang, D.T.; Chernomor, O.; von Haeseler, A.; Minh, B.Q.; Vinh, L.S. UFBoot2: improving the ultrafast bootstrap ap-proximation. *Mol. Biol. Evol.* **2018**, *35*, 518–522.

27. Katoh, K.; Rozewicki, J.; Yamada, K.D. MAFFT online service: multiple sequence alignment, interactive sequence choice and visualization. *Briefings Bioinform.* **2019**, *20*, 1160–1166, doi:10.1093/bib/bbx108.
28. Letunic, I.; Bork, P. Interactive Tree of Life (iTOL) v4: recent updates and new developments. *Nucleic Acids Res.* **2019**, *47*, W256–W259, doi:10.1093/nar/gkz239.
